# Cube natural sea salt ameliorates obesity in high fat diet-induced obese mice and 3T3-L1 adipocytes

**DOI:** 10.1038/s41598-020-60462-z

**Published:** 2020-02-25

**Authors:** Eui-Seong Park, Ting Yu, Kiho Yang, Shinil Choi, Seung-Min Lee, Kun-Young Park

**Affiliations:** 1 Department of Food and Nutrition, Yonsei University, Seoul, 03722 South Korea; 2 Department of Food Science and Biotechnology, Cha University, Seongnam, Gyeonggi-do 13488 South Korea; 3 Department of Oceanography, Pusan National University, Busan, 46241 South Korea; 4 DOCHO Agricultural Co., Ltd., Shinan, Jeollanam-do 58851 South Korea

**Keywords:** Marine biology, Obesity

## Abstract

Sodium is an essential component of the human body, with known influences on obesity. This paper reports the effect of cube natural sea salt (CNS) on the reduction of obesity in high fat diet-induced obese C57BL/6 mice and 3T3-L1 adipocytes, by ameliorating the obesity parameters and obesity-related gene mechanisms. The suppression of high fat diet-induced obesity and differentiated 3T3-L1 adipocytes by sea salt depends on the manufacturing process and mineral content. The manufacturing method using only new sea water (Cube natural sea salt) decreases the magnesium (Mg) and sulfur (S) content in the salt with different crystallization and morphologies, compared to the general manufacturing method (Generally manufactured sea salt, GS). Mg in salt is known to considerably affect obesity; an appropriate concentration of magnesium chloride (MgCl_2_) reduces lipid accumulation significantly and regulates the lipogenesis and liver enzyme activity. Our results indicate that sea salt contains an appropriate level of Mg as compared to table salt (purified salt, NaCl), and is important for regulating obesity, as observed in the *in vivo* and *in vitro* anti-obesity effects of CNS. The Mg content and mineral ratio of sea salt are important factors that ameliorate the lipid metabolism and liver enzyme activity in high fat diet induced obesity, and contents of Mg in sea salt can be altered by modifying the manufacturing process.

## Introduction

Salt is required to maintain homeostasis in the body by regulating the membrane potential, fluid volume, acid–base balance, and nervous system. Globally, many countries predominantly eat purified (iodized), rock, and sea salt. Purified salt (99% sodium chloride, NaCl; generally called table salt) is made from sodium ion (Na^+^) and chloride ion (Cl^−^) from sea water using an ion exchange membrane electrodialysis process, and is composed of more than 99% NaCl^1^. Sea salt (SS) is crystallized from seawater in salt ponds using sunlight and wind^2^. Unlike purified salt, SS generally contains 92.4~94.4% NaCl and various other minerals, such as potassium (K), magnesium (Mg), calcium (Ca), and sulfur (S)^3^. This imparts numerous health functionalities to SS^4–9^ and a unique taste^10^ as compared to purified salt. In addition, our previous study showed that the crystallization time and evaporating method of seawater are important factors affecting the quality, mineral composition, taste, and health functions of SS.

High salt consumption has synergistic effects in western diet-induced metabolic diseases^11^. In Europe and Northern America, most people eat processed foods (added high salt content using purified salt) compared to home cooked foods (as high as 75% in USA and UK)^12^. Moreover, high-income English-speaking countries have the highest rate of obesity in the world^13^. In addition, beverage consumption has increased the obesity risk^14,15^ because of their salt (purified salt) and sucrose content. Overall, one possible reason for obesity is the high intake level of purified salt^16^, which is cheaper than naturally manufactured salt. Therefore, most manufactured beverages and foods include purified salt, and most people unwittingly add purified salt to their diet. Dietary foods (purified salt consumption) are associated with the incidence of obesity, and purified salt intake has also been reported to increase the incidence of obesity compared to naturally manufactured salt^7^. It has previously been reported that SS can help to prevent obesity in high fat diet-induced obese mice, indicating that the consumption of SS may help reduce the incidence of obesity^7^.

Other studies have also reported that SS can help to prevent cancer^4–6^, obesity^7^, diabetes^8^, and hypertension^9^, as compared to purified salt (NaCl). Generally, the minerals (except NaCl) in SS include various cations, such as Mg, Ca, K, zinc (Zn), and iron (Fe)^3^. Few studies have reported the relationship between minerals present in the salt composition and obesity. In our previous studies of interaction between SS intake and obesity^7,17^, the Na:K ratio and Mg content in SS correlate with obesity in mice^7^ and 3T3-L1 adipocytes^17^. These results suggest that the beneficial health effects of SS are due to the presence of various minerals.

Mg is an essential cation essential for human homeostasis and supporting the physiological functions in the heart, brain, and skeletal muscles. The daily intake of Mg, as suggested by the United States Food and Nutrition Board is 420 mg and 320 mg for men and women, respectively^18^. On the other hand, current reports estimate that only 40% of Americans consume the required daily amount^19^. Several studies have indicated an association between the body mass index (BMI) and Mg intake status in humans^20–23^, but because these results are controversial^24^, the relationship between obesity and Mg remains unclear^25^.

Depending on their manufacturing process, salt crystals differ in shape. NaCl is produced using an ion exchange membrane and subsequently blended to a powder, whereas SS is crystallized by the direct evaporation of sea water, using sunlight and wind. The method of sea water crystallization is an important step^26^. Typically, manufactured SS is a mixture of old- and new seawater during evaporation (generally manufactured sea salt, GS), whereas another process using only new sea water during evaporation produces cube natural sea salt (CNS). This results in differing sizes, shapes, textures, and mineral contents of Mg and S.

## Results

### CNS reduces obesity in azoxymethane/dextran sodium sulfate + high fat diet induced colorectal cancer and obesity on C57BL/6 mice

**Figure 1:**
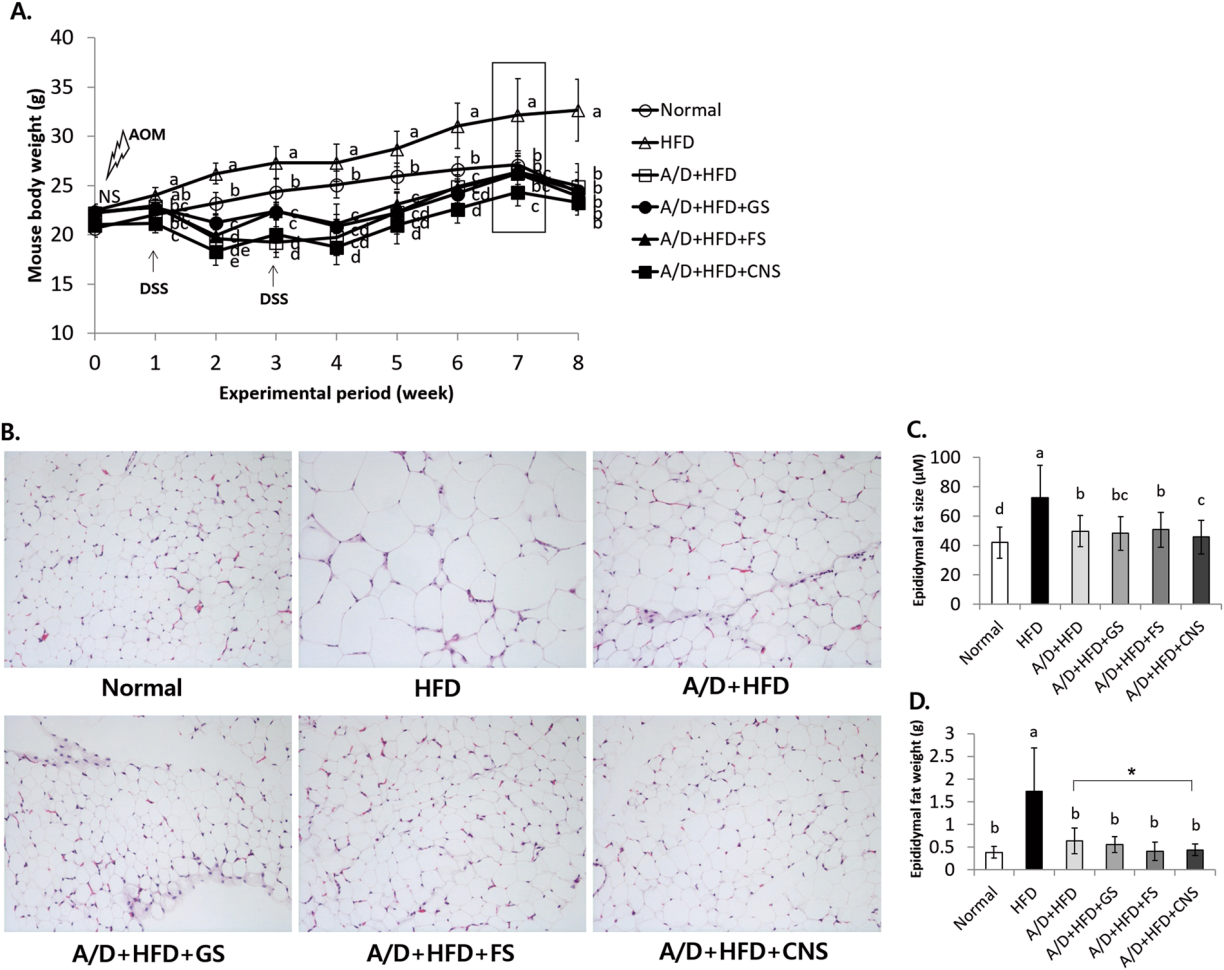
Cube natural sea salt (CNS) intake reduces mouse body weight, as well as number of lipid droplets and weight of epididymal adipose tissue. **(A)** Mouse body weight and histological analysis of epididymal adipose tissue and fat size, and body weight of C57BL/6 mice treated with A/D, HFD, and A/D + HFD. **(B)** Epididymal adipose tissue was observed using a light microscope following H&E staining **(C)** Epididymal adipose tissue size and **(D)** weight are shown. ^a–d^Means with different letters on the bars are significantly different (*P* < 0.05) by Duncan’s multiple range test. Magnification × 100.

As presented in Fig. 1A, the dextran sodium sulfate treatment reduces the weight of mice at two and four weeks. At seven weeks, the weight of the mice was observed to be the highest in the high fat diet group (32.2 ± 3.7 g), whereas the Normal (27.1 ± 1.2 g), A/D + HFD (azoxymethane (A)/dextran (D) + high fat diet (HFD)) (26.4 ± 0.9 g), A/D + HFD + GS (26.3 ± 1.7 g), and A/D + HFD + FS (FS, filtering sea water after evaporating sea salt) (26.2 ± 1.1 g) groups showed similar weights. The A/D + HFD + CNS group (24.3 ± 1.0 g) showed significantly lower body weight than the A/D + HFD group (Fig. 1A). At eight weeks (after fasting, except HFD), all mice body weights were similar, except for the HFD group. The normal group had a significantly different average food intake (AFI) compared to the other groups (Supplement Fig. 1A). The A/D + HFD-treated groups had a significantly different AFI compared to the HFD group at two and four weeks during the DSS treatment period, whereas at three and five-six weeks, the A/D + HFD groups were similar to the others. The normal group had a significantly increased food conversion ratio (FCR) compared to the HFD group at six and seven weeks (Supplement Fig. 1B). At two and four weeks, the standard deviations of the FCRs of the A/D + HFD as well as A/D + HFD + GS, FS, and CNS groups were large, and the FCR increased because the body weight gain value was negative upon the administration of D. At three and five-seven weeks, however, the group treated with A/D did not show a significant difference overall. The liver, testis, and kidney weights were similar among the groups (data not shown). Therefore, the A/D + HFD + CNS group had the lowest body weight at seven weeks, and 1% sea salt intake had no effect on AFI or FCR in mice.

The consumption of a HFD is associated with an increased size and number of adipocytes^27^. The epididymal fat size of all mice fed HFD was larger than that of normal mice. The CNS-treated mice showed a significantly reduced fat size and inflammation compared to the A/D + HFD group (Fig. 1B,C) as well as a significantly reduced epididymal fat weight compared the A/D + HFD group (*P* < 0.05) (Fig. 1D). Therefore, the CNS treatment reduced the fat size and weight in the fat and liver tissues as well as the body weight of A/D + HFD-induced mice.

**Figure 2:**
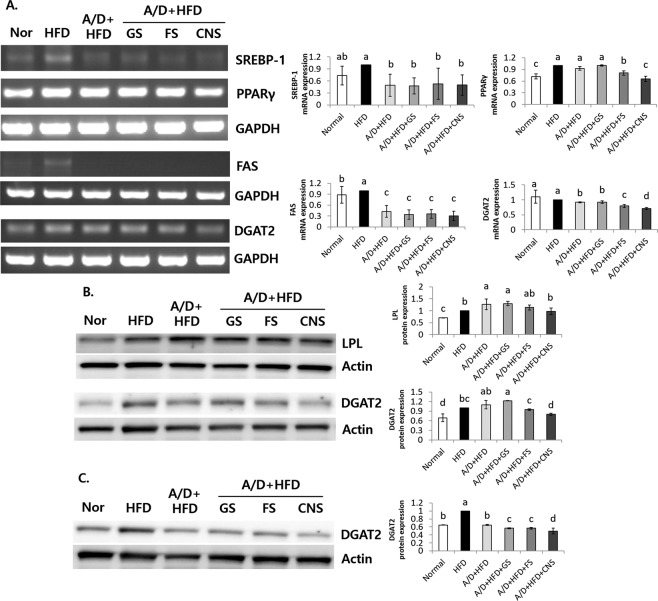
CNS regulates obesity related genes in A/D + HFD induced mice. **(A)** Adipo/lipogenesis related genes of mRNA expression levels of SREBP-1, PPARγ, FAS, and DGAT2 in liver tissue. **(B)** protein expression levels of the lipogenesis genes LPL and DGAT2 in the liver and **(C)** epididymal fat. Fold ratio: Gene expression/(GAPDH or Actin) × HFD numerical value (HFD fold ratio = 1). ^a–d^Means with different letters are significantly different (*P* < 0.05) by Duncan’s multiple range test. The grouping of gels/blots cropped from different gels/blots.

The sterol regulatory element-binding protein 1 (SREBP-1) and fatty acid synthase (FAS) expression levels were elevated in the HFD group and significantly lower in all groups treated with A/D compared to the HFD group (Fig. 2A). On the other hand, in the case of peroxisome proliferator-activated receptor gamma (PPARγ), the A/D + HFD and A/D + HFD + GS groups were similar to the HFD group, whereas the A/D + HFD + FS and A/D+HFD + CNS groups showed lower PPARγ expression than the HFD group. In particular, the A/D + HFD + CNS group showed the lowest level of PPARγ mRNA expression, similar to that of the Normal group (Fig. 2A). The A/D-treated groups also showed lower diglyceride acyltransferase 2 (DGAT2) mRNA expression than the HFD group, and the A/D + HFD + CNS group showed the lowest DGAT2 expression (*P* < 0.05) (Fig. 2B). The lipoprotein lipase (LPL) and DGAT2 levels were up-regulated in the HFD group compared to the Normal group (Fig. 2B,C). The A/D + HFD and A/D + HFD + GS groups showed the highest LPL and DGAT2 protein expression levels. On the other hand, the A/D + HFD + CNS group showed significantly lower LPL and DGAT2 levels in the liver and epididymal fat tissues compared to the A/D + HFD and A/D + HFD + GS groups (*P* < 0.05), whereas DGAT2 expression was similar to the Normal group.

Overall, CNS intake reduces obesity compared to other types of sea salt in A/D + HFD induced mice. On the other hand, the A/D + HFD model is a dual disease-induced mouse model. In this model, the mouse body weight loss and reduction of obesity-related gene expression cannot be attributed directly to the sea salt or A/D treatment. Therefore, this study investigated the relationship between sea salt and obesity in 3T3-L1 adipocytes and HFD-induced obese mice.

### CNS treatment reduces obesity in 3T3-L1 adipocytes

The survival rate of the 1% sea salt groups was not reduced significantly (Supplement Fig. 2A). NaCl was not used in the experiment because of the significantly different cytotoxicity between NaCl and CNS (Supplement Fig. 2B). In addition, 1% CNS reduced the cell viability of differentiated 3T3-L1 adipocytes compared to other salts (Supplement Fig. 2C).

**Figure 3:**
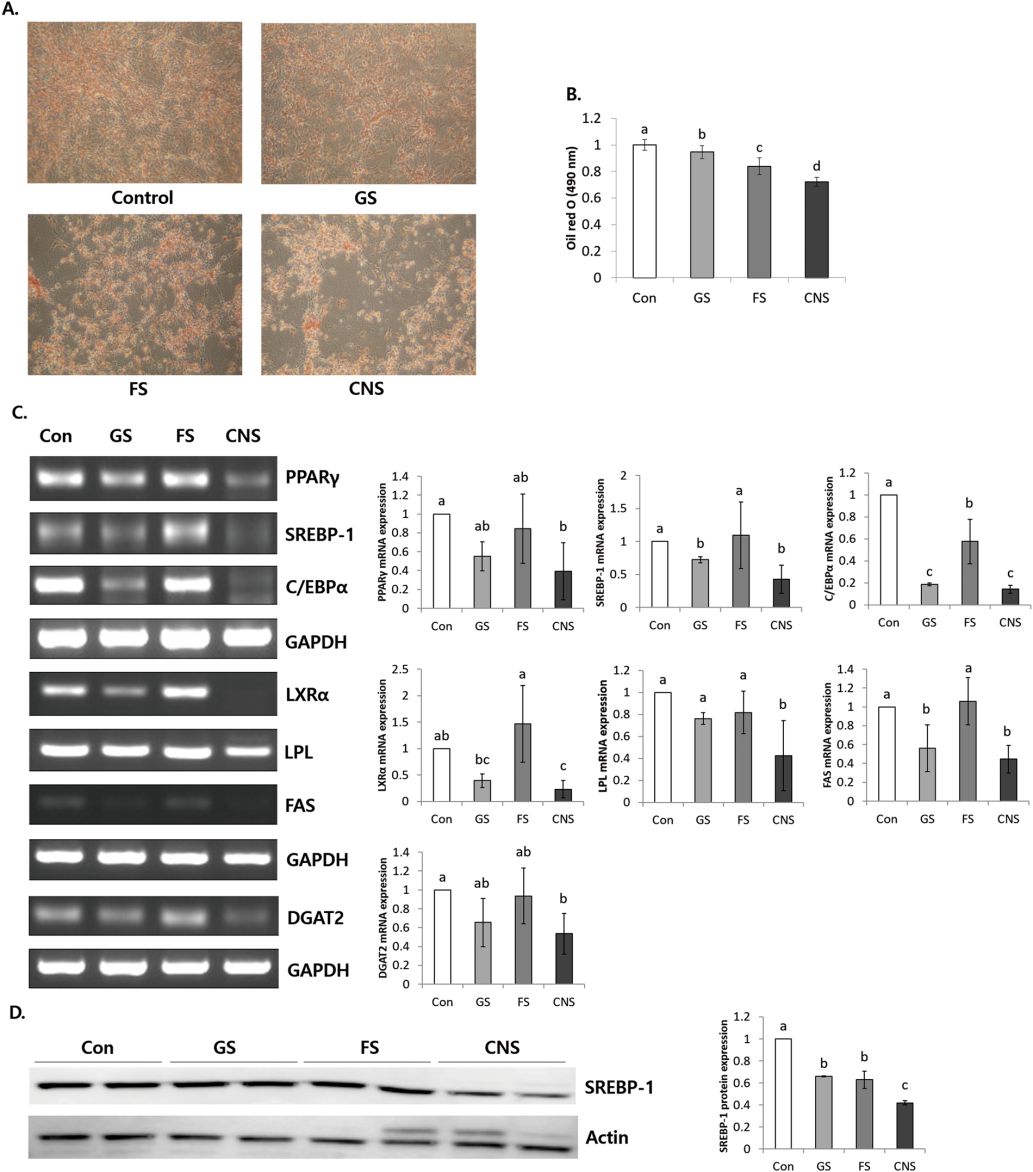
CNS treatment reduces lipid droplet and obesity related genes in differentiated 3T3-L1 adipocytes. **(A)** Oil red O staining, **(B)** 490 nm absorbance value, **(C)** mRNA expression levels of the adipo/lipogenesis-related genes PPARγ, SREBP-1, C/EBPα, LXRα, LPL, FAS, and DGAT2, and **(D)** protein expression levels of SREBP-1 in differentiated 3T3-L1 adipocytes. GS: Generally manufactured sea salt (mixture of concentrated old and new seawater) (1%), FS: Filtering processed sea salt (mixture of concentrated old and new seawater filtered through a charcoal and magnetic filter) (1%), CNS: Cube natural sea salt (sea salt made from only concentrated new seawater) (1%). Fold ratio: Gene expression/(GAPDH or Actin) × Control numerical value (Control fold ratio = 1). The grouping of gels/blots cropped from different gels/blots.

CNS resulted in a significant reduction of Oil red O-stained fat compared to the Control, GS, and FS (Fig. 3A,B). CNS resulted in significantly decreased mRNA expression levels of PPARγ (0.39 ± 0.3), SREBP-1 (0.42 ± 0.21), CCAAT-enhancer-binding proteins alpha (C/EBPα) (0.14 ± 0.04), liver X receptor alpha (LXRα) (0.22 ± 0.17), FAS (0.44 ± 0.15), DGAT2 (0.53 ± 0.21), and LPL (0.42 ± 0.32) in differentiated 3T3-L1 adipocytes compared to the Control (*P* < 0.05) (Fig. 3C). In addition, CNS resulted in significantly reduced levels of SREBP-1 protein expression (0.42 ± 0.02) compared to the Control (fold ratio: 1) (*P* < 0.05) (Fig. 3D). Therefore, CNS inhibits adipo/lipogenesis via the regulation of related gene expression.

### CNS intake reduces the basic parameters of obesity compared to NaCl and GS intake in high fat diet induced obese mice

**Figure 4:**
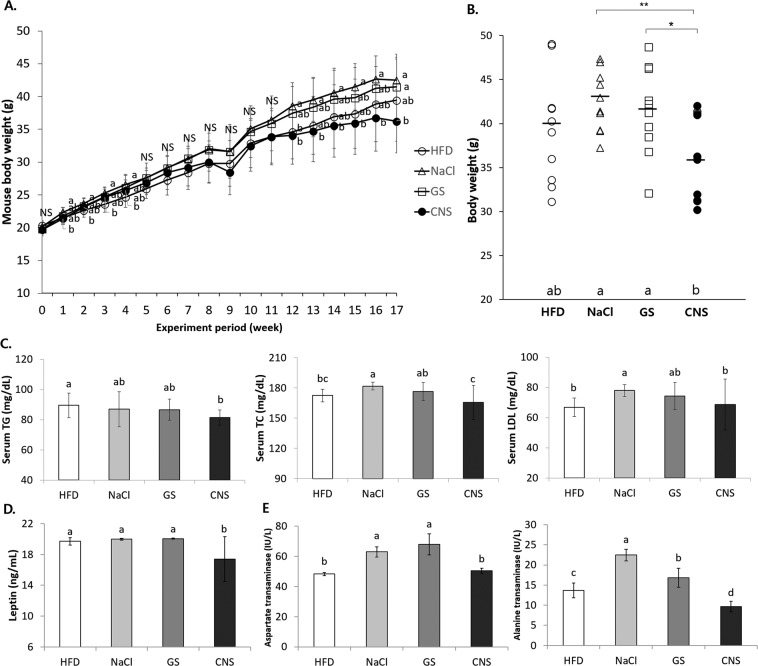
CNS intake reduces basic parameters of obesity in high fat diet induced obese mice. **(A)** Mouse body weight calculated at 0–17 weeks. **(B)** Mouse body weight at 17 weeks, expressed individually. **(C)** Lipid profiles of TG, TC, and LDL level in mice serum. **(D)** Levels of leptin, an obesity related hormone, in mice serum. **(E)** Liver enzyme activity of AST and ALT in mice serum. HFD: 45% high fat diet, NaCl: 45% high fat diet + NaCl (reagent) (1%), GS: 45% high fat diet + generally manufactured sea salt (mixture of concentrated old and new seawater) (1%), CNS: 45% high fat diet + Cube natural sea salt (sea salt made from only concentrated new seawater) (1%). ^a–c^Means with different letters are significantly different (*P* < 0.05) by Duncan’s multiple range test.

A high fat diet (HFD) group of animals gains more weight than those fed a normal diet. Because this study focused on the interaction between HFD and salt samples, only the HFD and HFD + sample treated groups were compared; a normal diet group was not included. As shown in Fig. 4A, a significant decrease in body weight was observed in the CNS treatment group as compared to the NaCl (Reagent, Sigma-Aldrich Co., St. Louis, MO, USA) group at 12–16 weeks (*P* < 0.05). At 17 weeks, the reduction in body weight was significantly different in the CNS group (36.2 ± 4.9 g) than both the NaCl group (42.5 ± 3.5 g) and GS group (41.5 ± 5.0 g) (*P* < 0.05) (Fig. 4B). No significant difference in the AFI was observed between the groups (Supplement Fig. 3A). On the other hand, the CNS group had a significantly higher FCR than the HFD group at 16 and 17 weeks (Supplement Fig. 3B). At nine weeks, all mice inadvertently showed a decrease in body weight with a reduced AFI and enhanced FCR. After nine weeks, however, the body weight, AFI, and FCR of all the mice returned to normal conditions.

The measured serum lipids profiles revealed a significant reduction of triglyceride (TG), total cholesterol (TC), and low density lipoprotein (LDL) in the CNS group as compared to the NaCl group (*P* < 0.05) (Fig. 4C). In addition, the CNS group showed significantly reduced leptin levels (a serum obesity related hormone) as compared to HFD, NaCl, and GS groups (*P* < 0.05) (Fig. 4D). The liver enzyme concentrations showed significant reduction of aspartate transaminase (AST) and alanine transaminase (ALT) in the CNS group as compared to the NaCl group (*P* < 0.05) (Fig. 4E). On the other hand, the Na concentrations and glucose level in the serum remained unaffected with all salt treatments (Supplement Fig. 4A,B), indicating that the type of salt has no effect on the glucose metabolism, and 1% salt intake does not influence blood homeostasis in mice.

These observations showed that the CNS group had the lowest body weight at 17 weeks. Although the AFI or FCR were unaffected by 1% NaCl and the sea salt intake, the CNS group showed enhanced FCR at 16 and 17 weeks. In addition, the serum examination revealed reduced lipid accumulation, leptin and liver enzymes in the CNS group. Overall, NaCl intake increases obesity, but the CNS intake results in decreased obesity compared to NaCl intake in HFD-induced obese mice.

### CNS intake regulates fatty liver, fat size, and expression of genes related to adipo/lipogenesis, β-oxidation, and lipolysis in liver and epididymal white adipose tissue in high fat diet induced obese mice

**Figure 5:**
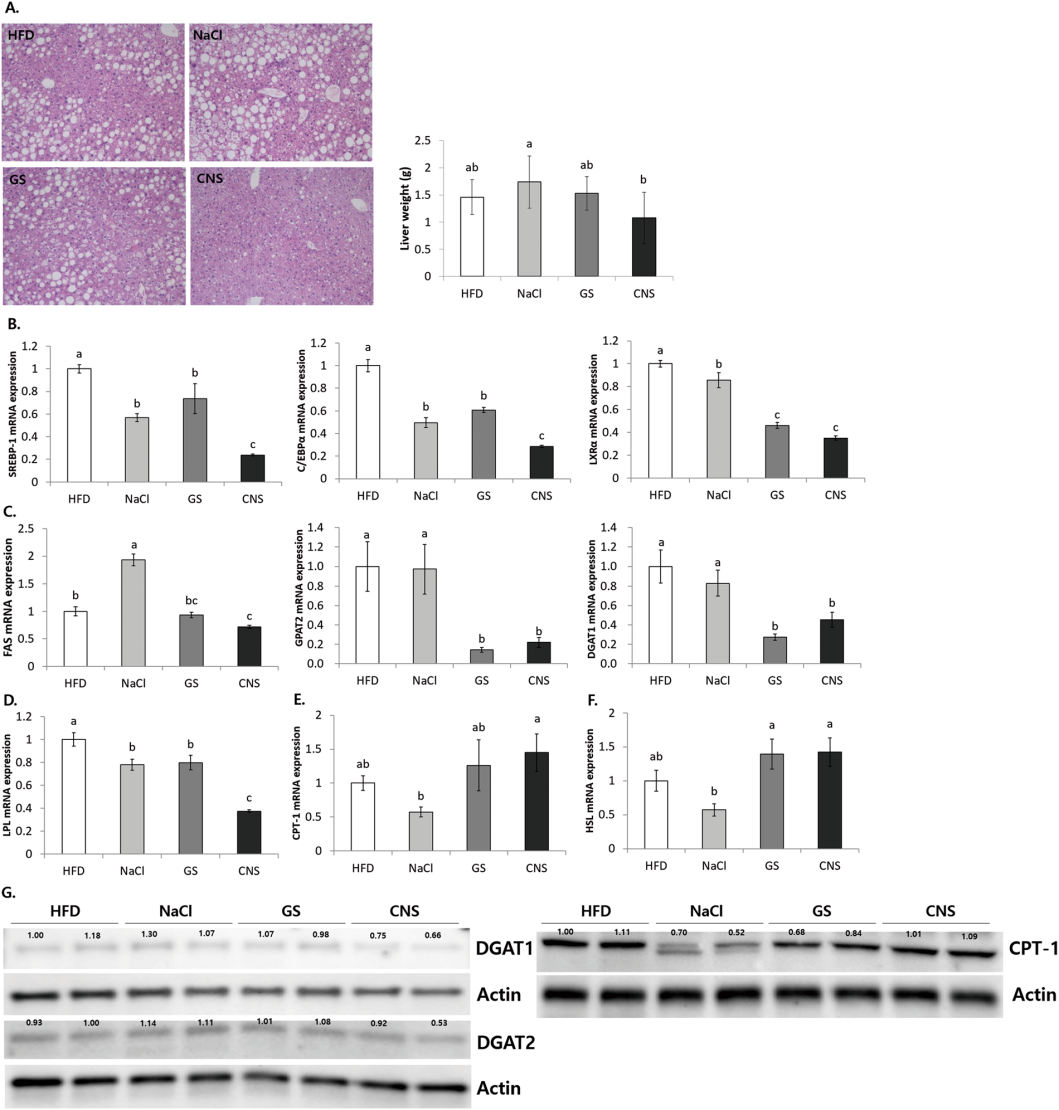
CNS intake reduces lipid droplet and regulates obesity related gene expression in liver tissue. **(A)** Histological observation of hematoxylin and eosin (H&E) stained liver tissue, and corresponding liver tissue weight. **(B–F)** mRNA expressions of: **(B)** adipo/lipogenesis related transcription factor genes, **(C)** triglyceride storage (lipogenesis) related genes, **(D)** gene related to regulation of triglyceride and cholesterol storage or release by chylomicron, **(E)** β-oxidation related gene, and **(F)** lipolysis related gene. **(G)** Protein expressions of lipogenesis and β-oxidation related genes. ^a–c^Means with different letters are significantly different (*P* < 0.05) by Duncan’s multiple range test. Gene expression/Actin × HFD numerical value (HFD fold ratio = 1) used ImageJ program. The grouping of blots cropped from different blots.

As shown in Fig. 5A, HFD induces the accumulation of lipid droplets and weight growth in liver tissue. The NaCl group had highly accumulated lipid droplets and increased number of inflammatory cells, loss of nuclei, and severe swelling of hepatocytes as compared to the other groups. The CNS group showed significantly reduced lipid droplets and number of inflammatory cells compared to the HFD, NaCl, and GS groups. The liver weight was significantly decreased in the CNS group (1.08 ± 0.47 g) as compared to the NaCl group (1.74 ± 0.48 g).

Considering the adipo/lipogenesis related transcription factors, namely, SREBP-1, C/EBPα, and LXRα, the CNS group showed significantly lower mRNA expression than the HFD and NaCl groups (*P* < 0.05) (Fig. 5B). In addition, the expression levels of the lipogenesis-related genes of FAS, glycerol-3-phosphate acyltransferase 2 (GPAT2), DGAT1, and LPL were also significantly reduced in the CNS group when compared to HFD and NaCl groups (*P* < 0.05) (Fig. 5C,D). In particular, the CNS group (1.44 ± 0.28) showed higher levels of mRNA expression of a β-oxidation related gene, carnitine palmitoyltransferase 1 (CPT-1), than the NaCl group (0.57 ± 0.07) (Fig. 5E). Furthermore, the CNS group (1.42 ± 0.21) also showed enhanced levels of the lipolysis-related gene of hormone sensitive lipase (HSL) compared to the NaCl group (0.57 ± 0.09) (*P* < 0.05) (Fig. 5F). The protein levels of DGAT1 and 2, were lower, whereas the CPT-1 levels were higher in the CNS group (1.01 ± 0.01) than the NaCl group (0.61 ± 0.13) (*P* < 0.05) (Fig. 5G). Considering adipo/lipogenesis, the NaCl group showed significantly increased FAS compared to the HFD group, but in β-oxidation and lipolysis, the NaCl group markedly showed decreased CPT-1 and HSL compared to the HFD group. These results indicate that consumption of NaCl may suppress the expression of β-oxidation and lipolysis in the liver tissue, thereby enhancing obesity by improving the levels of lipid accumulation and body weight gain. On the other hand, CNS intake inhibited adipo/lipogenesis and promoted β-oxidation and lipolysis via the regulation of related gene expression compared to the HFD and NaCl groups, and showed better suppression of obesity.

**Figure 6:**
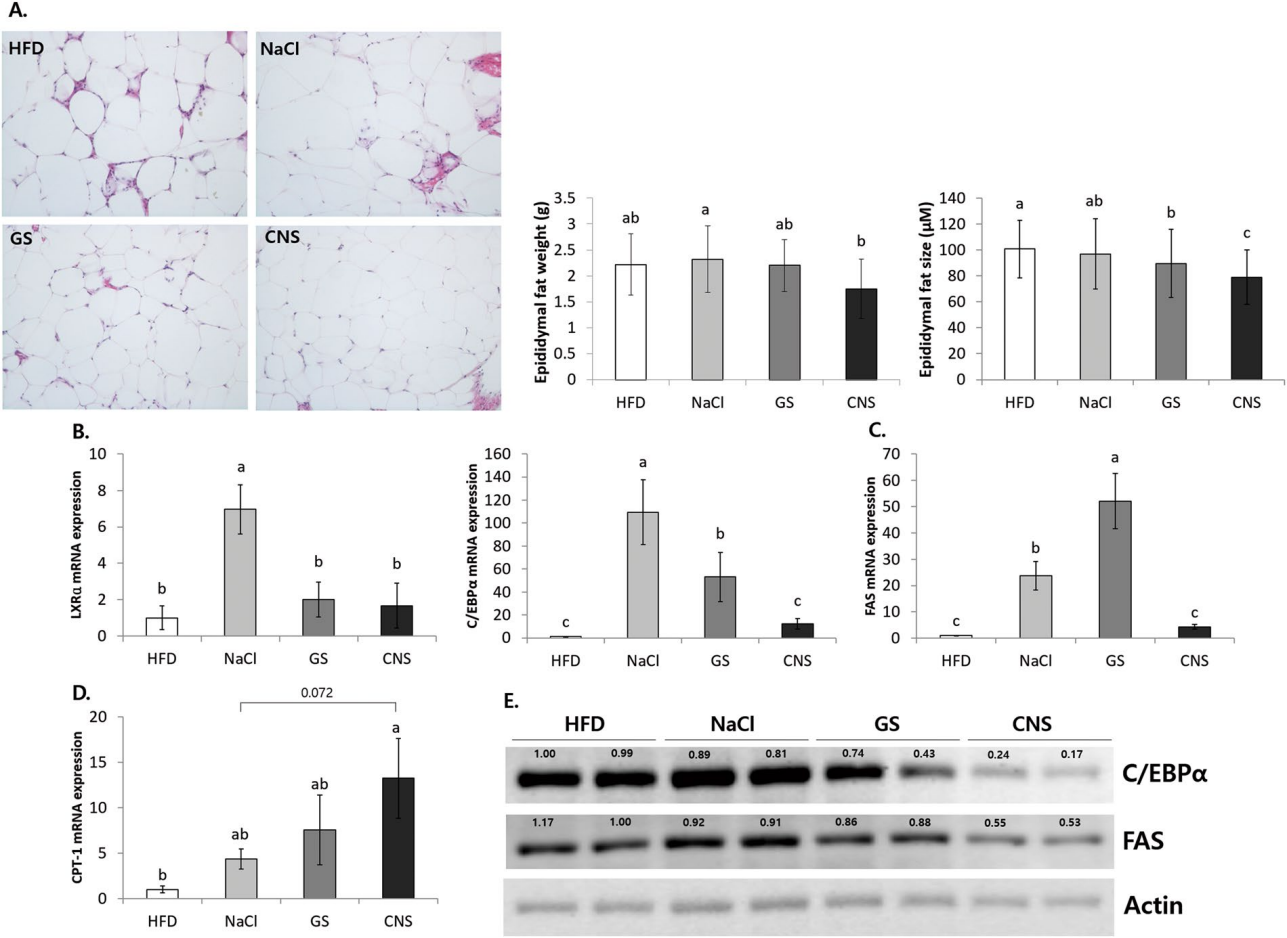
CNS intake decreases fat size and weight, and regulates obesity related gene expression in epididymal white adipose tissue. **(A)** Histological observation of epididymal fat tissue using hematoxylin and eosin (H&E) staining, and corresponding weight and size of fat tissue. **(B–D)** mRNA expressions of **(B)** adipo/lipogenesis related transcription factor genes, **(C)** triglyceride storage (lipogenesis) related genes, and **(D)** β-oxidation related gene. **(E)** Protein expressions of adipo/lipogenesis related genes. ^a–c^Means with different letters are significantly different (*P* < 0.05) by Duncan’s multiple range test. Gene expression/Actin × HFD numerical value (HFD fold ratio = 1) used ImageJ program. The grouping of blots cropped from different blots.

As shown in Fig. 6A, HFD induced an increase in the amounts of fat in the epididymal white adipose tissue of mice. The HFD and NaCl groups showed a significantly increased fat size, number of inflammatory cells, loss of nuclei, and crown-like structures compared to the other groups. The CNS group (1.75 ± 0.57 g) had significantly lower epididymal white adipose tissue weight as compared to the NaCl group (2.32 ± 0.64 g). Moreover, the CNS group (79.0 ± 20.9 μm) also showed significantly reduced epididymal white adipocyte size as compared to the NaCl (96.8 ± 27.1 μm) and HFD (100.7 ± 22.2 μm) groups.

**Figure 7:**
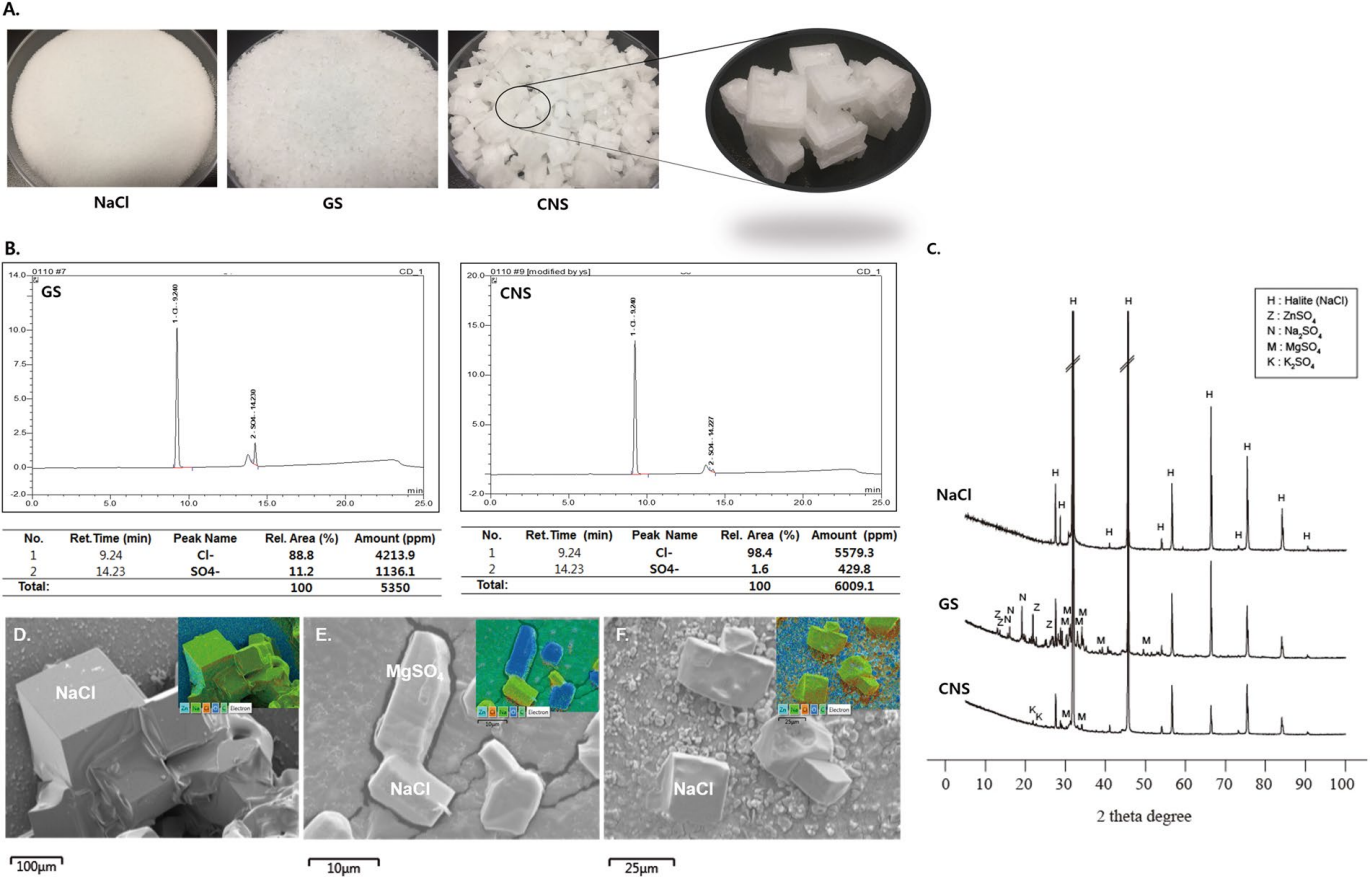
Different manufactured salts have different anion concentration, mineral assemblage, and morphology. **(A)** Appearance of differently manufactured salts. **(B)** Anion concentration of three types of solar salts (NaCl, GS, and CNS) by using ion chromatograph (IC) analytical instrument. Anions of two elements (Cl and SO_4_) were measured (units: ppm). **(C)** X-ray diffractogram (XRD) profiles for air dried solar salts (NaCl, GS, and CNS) displaying composition of halite (NaCl), and sulfate mineralized salts of zinc sulfate (Z), sodium sulfate (N), magnesium sulfate (M), and potassium sulfate (K). All three samples show prominent halite (NaCl) peak (31.82°, 3.11Å and 45.541°, 1.99Å). **(D–F)** SEM micrograph of mineralized solar salt structures and inset images of EDS elemental distribution maps. **(D)** Aggregates of high crystalline of cubic structure of halite (NaCl) in control. **(E)** Randomly distributed elongated shaped poorly crystalline halite and MgSO_4_ ~10 µm in length in GS. **(F)** Underdeveloped cubic structure of halite ~25 µm with sulfate minerals of few nanometer size in CNS.

In the CNS group significantly decreased mRNA expression levels of the adipo/lipogenesis related transcription factors, LXRα and C/EBPα, were observed compared to the NaCl group (*P* < 0.05) (Fig. 6B). Furthermore, the lipogenesis related genes of FAS also showed significantly reduced mRNA expression in the CNS group as compared to NaCl group (*P* < 0.05) (Fig. 6C). The CNS group promoted the mRNA expression of the β-oxidation related gene, CPT-1 (13.23 ± 4.40), compared to the NaCl group (4.35 ± 1.09) (*P* = 0.072) (Fig. 6D). Regarding the protein levels, the CNS group showed significantly decreased protein levels of C/EBPα and FAS when compared to the NaCl group (Fig. 7E). In addition, the NaCl group revealed significantly increased mRNA expression of LXRα, C/EBPα, and FAS, but decreased protein levels of C/EBPα and FAS as compared to the HFD group. As indicated previously, NaCl decreases the mRNA expression of transcription factors of adipo/lipogenesis related genes in the liver (Fig. 5B). In particular, the feces lipid levels remained unaffected by all salt treatments (Supplement Fig. 5). Therefore, NaCl intake has a strong influence on β-oxidation and lipolysis, as against adipo/lipogenesis in the mouse liver. The reduced fat oxidation and lipolysis due to NaCl intake may therefore be associated with the accumulation of excess fat. However, sea salt (GS and CNS) intake inhibits adipo/lipogenesis and promotes β-oxidation in epididymal white adipose tissue when compared to NaCl, and especially, CNS intake was seen to be more efficacious than GS.

### Manufacturing process of salts affects the cation, anion, assemblage, and morphology

The chemical composition of dissolved sea salt samples (GS and CNS) revealed different elemental concentrations of cations when compared to the NaCl (Table 1). High concentrations of Mg and S with low concentrations of Fe were present in the sea salt samples as compared to the NaCl of control sample. Both Cu and P were absent. Different concentrations of cations were present in the two types of sea salt (GS and CNS). GS contained more Mg, Ca, S, Zn, and Fe (5.3, 1.6, 9.4, 1.1, and 3.6 times, respectively) as compared to CNS, whereas Na and K contents were lacking (less than 0.77% and 0.87%, respectively).

**Table 1:** Elemental concentration of three types of solar salt samples (NaCl, GS, and CNS) evaluated by the ICP-OES analytical instrument.

	NaCl	GS	CNS
Na (g/kg)	332.86 ± 3.51^a^	225.56 ± 5.66^c^	294.57 ± 3.28^b^
Mg (g/kg)	Not detected	41.83 ± 0.20*	7.96 ± 0.10
K (g/kg)	5.59 ± 0.18^c^	8.39 ± 0.02^b^	9.63 ± 0.24^a^
Ca (g/kg)	Not detected	1.42 ± 0.02*	0.91 ± 0.00
S (g/kg)	Not detected	10.21 ± 0.16*	5.00 ± 0.03
Zn (mg/kg)	0.34 ± 0.02^NS^	0.32 ± 0.03	0.30 ± 0.02
Fe (mg/kg)	Not detected	1.10 ± 0.06*	0.30 ± 0.03
Cu (mg/kg)	Not detected		

Nine cations of elements (Na, Mg, K, Ca, S, Zn, Fe, CU, and P) were measured (units: ppm). ^a–c^Means with different letters are significantly different (*P* < 0.05) by Duncan’s multiple range test. *<0.05 student t-test (between GS and CNS).

The appearance of salts varies according to their processing. In this study, the three types of salts had different appearances. In particular, the shape of CNS was cubic, which is produced naturally (Fig. 7A).

The analysis results of all three samples showed that the Cl^−^ content was higher than SO_4_^2−^. GS had lower Cl^−^ than CNS (4214 ppm and 5579 ppm, respectively), whereas SO_4_^2−^ content was higher (1136 ppm and 430 ppm, respectively) (Fig. 7B).

The X-ray diffractometer (XRD) analysis profiles were recorded using the Cu-K X-ray source (λ = 1.5406 Å). Profiles were recorded while rotating the randomly oriented samples, enabling us to check the rotation angle during diffraction. Signals were interpreted into diffraction peaks in the XRD profiles^28^. XRD analyses of salts (NaCl, GS, and CNS) were performed for the different manufacturing procedures (Fig. 7C). NaCl of the control sample was examined to confirm the purity of NaCl and KCl. The heterogeneous suite of minerals identified by XRD in GS includes ZnSO_4_, K_2_SO_4_, Na_2_SO_4_, MgSO_4_, and NaCl. The minerals identified in CNS include NaCl, K_2_SO_4_, and MgSO_4_, with NaCl peaks appearing more prominently as compared to GS. The discernible sulfate mineral XRD reflections were not observed due to the lack of sulfate, as confirmed by inductively coupled plasma-optical emission spectrometry (ICP-OES) (Table 1).

Scanning electron microscopy (SEM) of the SS samples from GS, CNS, and control showed that the NaCl mineral typically appeared as aggregates of well-developed euhedral cubic structures (Fig. 7D), clusters of randomly distributed square columnar structure with varied sizes (~10 µm) which are confirmed as MgSO_4_ of sulfate minerals and NaCl (GS, Fig. 7E), and relatively bigger size crystals of NaCl (~25 µm) that appear to be underdeveloped and were identified to be devoid of the prominent shape of sulfate minerals (CNS, Fig. 7F).

### An appropriate concentration of MgCl_2_ is required to regulate obesity

Seawater contains numerous ionized minerals. Because sea salt is produced by the evaporation of seawater, the difference in crystallinity of the constituent minerals varies significantly, depending on the environment in which the minerals are precipitated, including the presence of impurities and the time for recrystallization^29^. We previously demonstrated the presence of NaCl, KCl, ZnSO_4_, K_2_SO_4_, Na_2_SO_4_, and MgSO_4_ in NaCl (reagent), GS, and CNS (Fig. 7). However, during consumption, most minerals in salt are dissolved with water and are ionized (Supplement Fig. 6) to release Na^+^, K^+^, Ca^2+^, Mg^2+^, Cl^−^, and SO_4_^2−^ ^30^, which in turn influence the body. Evaluation of the mineral ions revealed that the Cl^−^ content in CNS was 13 times higher than SO_4_^2−^ (Fig. 4B). Different concentrations of MgCl_2_ based on the sea salt Mg contents were selected considering that the aim was to evaluate the effects of Mg^2+^ on regulating obesity with the Cl^−^ content being a default.

**Figure 8:**
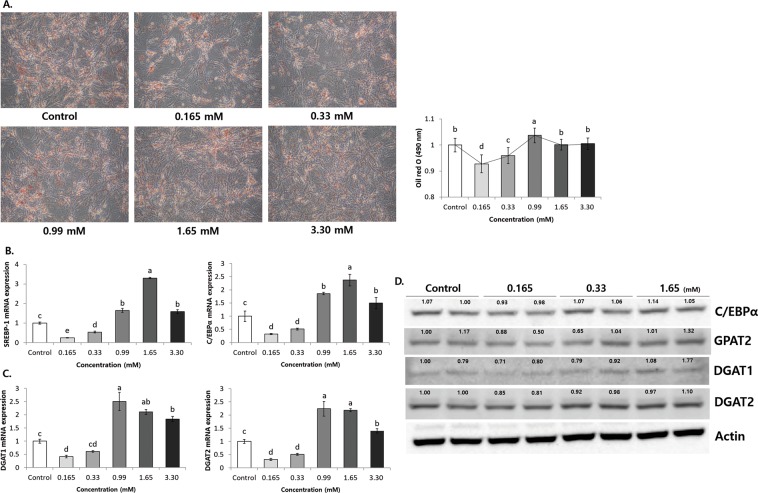
Appropriate concentrations of MgCl_2_ reduces lipid droplet accumulation and adipo/lipogenesis related gene expression in differentiated 3T3-L1 adipocytes. **(A)** Microscopic observation and optical density (OD, 490 nm) of different concentrations of MgCl_2_ treated 3T3-L1 cells. **(B)** mRNA expression of adipo/lipogenesis related genes of transcription factors. **(C)** mRNA expression of triglyceride storage (lipogenesis) related genes. **(D)** Protein expression of adipo/lipogenesis related genes. ^a–c^Means with different letters are significantly different (*P* < 0.05) by Duncan’s multiple range test. Gene expression/Actin × Control numerical value (Control fold ratio = 1) used ImageJ program. The grouping of blots cropped from different blots.

In this study, 3T3-L1 adipocytes were exposed to various concentrations of MgCl_2_. We observed that 0.165 mM and 0.33 mM MgCl_2_ significantly decreased lipid droplets in 3T3-L1 adipocytes (Supplement Fig. 7). As shown in Fig. 8A, 0.165 mM markedly reduced the lipid droplets, accumulated lipid, and Oil red O stained lipids considerably. Furthermore, exposure to 0.165 mM resulted in significantly reduced the mRNA expression levels of adipo/lipogenesis related transcription factors SREBP-1 (0.25 ± 0.02), C/EBPα (0.32 ± 0.02), and LXRα (0.59 ± 0.08) as compared to the Control (fold ratio: 1) (*P* < 0.05) (Fig. 8B). However, 0.99 mM, 1.65 mM, and 3.30 mM significantly increased the mRNA expression levels of SREBP-1 and C/EBPα, as compared to Control. Exposure to 0.165 mM also resulted in significant decrease in the lipogenesis related genes of mRNA expression levels of DGAT1 (0.41 ± 0.05) and DGAT2 (0.31 ± 0.04), as compared to the Control (fold ratio: 1) (*P* < 0.05) (Fig. 8C), whereas 0.99 mM, 1.65 mM, and 3.30 mM tended to increase the mRNA expression levels significantly. Assessing the levels of protein expression, 0.165 mM significantly decreased C/EBPα, GPAT2, DGAT1, and DGAT2, but 1.65 mM significantly increased these genes compared to the Control (*P* < 0.05) (Fig. 8D). In addition, 0.33 mM showed a similar tendency to 0.165 mM. Taken together, these results suggest that the appropriate MgCl_2_ concentrations regulate in reducing obesity, but MgCl_2_ concentrations beyond this optimal range induce obesity by regulating the adipo/lipogenesis related genes in 3T3-L1 adipocytes.

### MgCl_2_ inhibits ALT enzyme activity

**Figure 9:**
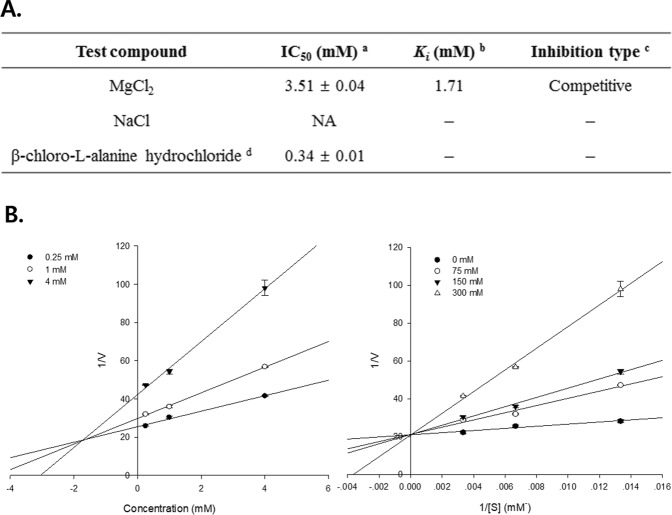
MgCl_2_ inhibits ALT enzyme activity to regulate competitive substrate and enzyme linkage. **(A)** ^a^Test concentrations of these compounds were in the range of 0.125–4 mM, dissolved in ALT assay buffer: 50% inhibition concentrations (IC_50_, mM) are expressed as the mean ± S.D. of triple experiments. ^b^The inhibition constant (*K*_*i*_) was determined by interpretation of the Dixon plot. ^c^Determined by interpretation of the Lineweaver-Burk plot. ^d^Used as positive control in assay. NA: no activity. **(B)** Dixon and Lineweaver-Burk plots of the inhibition of ALT by MgCl_2_. The result presented demonstrates the effects of the presence of different concentrations of the substrate for MgCl_2_, and the effects of the presence of different concentrations of MgCl_2_.

Results of this study reveal that CNS intake decreases the AST and ALT enzyme activity in HFD-induced obese mice serum (Fig. 4E), and MgCl_2_ reduces the lipid accumulation in 3T3-L1 adipocytes (Fig. 8). This study hypothesized that Mg intake will affect obesity and liver enzyme activity, and therefore examined the interaction between Mg and ALT enzyme activity. The IC_50_ values were evaluated to identify the inhibitory potential of MgCl_2_ and NaCl against ALT by performing the ALT inhibitory assay using L-alanine as the substrate. MgCl_2_ was effective in inhibiting the ALT activity in a concentration-dependent manner with an IC_50_ value of 3.51 ± 0.04 mM, when compared to the positive control (0.34 ± 0.01 mM) (Fig. 9A), but NaCl did not exhibit any ALT inhibitory activity. Enzyme kinetic analysis was performed to explain the mode of enzymatic inhibition with various concentrations of the corresponding substrate for ALT with the test sample (MgCl_2_). As shown in Fig. 9B, increasing the concentration of the substrate intersects the *y*-axis in the Dixon plot, indicating that MgCl_2_ competes with the substrate to bind to the active site of the ALT enzyme with a low *K*_*i*_ value of 1.71 mM, suggesting that MgCl_2_ has significant ALT inhibitory activity.

## Discussion

Many countries have recently recommended reduced salt consumption for improving human health. However, most research has focused on reducing the level of salt consumption. On the other hand, there has been little research on the NaCl salt type and diseases. Therefore, this study focused on the obesity regulation between SS containing various minerals and table salt.

Generally manufactured SS (GS) is a mixture of old and new seawater used during the process of evaporation in natural sunlight and wind, whereas CNS production uses only new seawater and has reported better health benefits compared to purified salt (NaCl) and other kinds of SS^6,7^. Compared to GS and FS, CNS reduces the mouse body weight, as well as size, weight, and number of lipid droplets in the epididymal fat tissues, and also inhibits expressions of the adipo/lipogenesis-related genes in A/D + HFD induced colorectal cancer and obesity mice model. This indicates the differing characteristics of SS according to the methods of processing, thereby exerting different health benefits. Since the A/D + HFD model is a dual disease-induced mouse model, mouse body weight loss and reduction of obesity-related gene expression cannot be attributed directly to sea salt or A/D treatment. This study therefore investigated the obesity-related factors in 3T3-L1 adipocytes.

LXRα activates transcription factors, and specifically plays a general role in lipid metabolism^31^. SREBP-1^32^ and C/EBPα^33^ induced by LXRα activates acetyl-CoA carboxylase (ACC), FAS, and stearoyl-CoA desaturase 1 (SCD-1); these genes subsequently regulate accumulation of fatty acids and TG^34^. The enzyme LPL regulates energy balance and body weight as well as catalyzes hydrolysis of TG, lipoproteins, chylomicrons, and very low-density lipoprotein (VLDL)^35^. CNS reduces the expressions of LXR, C/EBPα, and SREBP-1. Since LXR regulates various genes related to cholesterol and fatty acid metabolism in the liver^31^, inhibition of 3T3-L1 lipid accumulation can be attributed to the regulation of genes associated with adipo/lipogenesis. In this study, only CNS treatment reduced the number and size of lipid droplets in fat tissue and 3T3-L1 adipocytes, as well as inhibited adipo/lipogenesis related gene expression, when compared to GS. However, although the anti-obesity effects of CNS were confirmed, there was no comparative study between CNS and NaCl. Thus, NaCl and CNS were given at the same concentrations, and compared and confirmed in the next experiment.

Many studies have reported that salt intake is strongly related to obesity in humans^36,37^ and mice^38^. In this study, 1% NaCl intake increased the mouse body weight, but CNS intake decreased the same, and these results are similar to our previous studies. This has been clarified in other experiments using 1% concentrated NaCl or SS intake^6,7^, and this concentration did not affect the blood sodium concentration in mice (Supplement Fig. 4A). Moreover, the mice were treated with 1% salts for 17 weeks, but no abnormal mouse behaviors or deaths were observed. Overall, 1% CNS intake decreased body weight of high fat induced obesity compared to NaCl, and 1% salt intake did not have any toxicity in the 17 weeks experiment period.

Obesity parameters include percentage of fat^39^, TG, TC^40^, ALT, AST^41^, leptin^42^, adipogenesis (SREBP-1, C/EBPα, PPARγ)^43^, and lipogenesis related genes (SREBP-1, C/EBPα, LXRα)^44^. Ju *et al*.^7^ reported that SS reduced the mouse body weight, epididymal white adipose tissue weight, TG, TC, and leptin in the serum of HFD-induced obese mice. Moreover, a deep seawater treatment also reduced the mouse body weight, TG, TC in *ob/ob* mice^45^. In the current study, all mice consumed the same amount of food, but the mice who ingested CNS with their high fat diet could lose weight, and improve their lipid profiles and leptin. Moreover, high fat diet induces non-alcoholic fatty liver disease (NAFLD), which refers to the accumulation of liver steatosis and is unrelated to excess alcohol consumption^46^. As excessive fat develops in the liver, it damages hepatic cells, which elevates the serum levels of ALT and AST enzyme activity in the blood^47^. The consumption of CNS regulated the lipid profiles, leptin, and liver enzyme activity with a resulting decrease in mouse body weight. Despite the same SS, however, GS was less effective in inhibiting obesity than CNS. Therefore, although the various minerals present in SS can be attributed to the regulation of obesity, an appropriate mineral composition is very important.

Interestingly, NaCl intake did not significantly affect the diabetes related factors of serum glucose (Supplement Fig. 4B). Hoffmann and Cubeddu^48^ reported that urinary sodium is related to body weight and BMI, but not fasting glucose. Therefore, NaCl intake increases obesity but is inconsequential for blood glucose.

High fat diet intake induces the production of a fatty acid pool and TG accumulation in liver tissue^49^. CNS intake decreased the lipid accumulation and weight in liver tissue compared to the NaCl intake group. These results indicate that CNS intake regulates the lipid metabolism in hepatic tissue. Adipogenesis is the process that transforms pre-adipocytes into mature differentiated adipocytes^43^, and lipogenesis is the process of fatty acid synthesis from acetyl‐CoA subunits^44^. In this study, CNS intake suppresses the transcription of both adipo/lipogenesis (PPARγ, C/EBPα, SREBP-1, and LXRα) and lipogenesis related genes (FAS, GPAT2, DGAT1, 2, and LPL). In particular, FAS, being a multifunctional enzyme in the lipogenesis pathway^44^, was not increased by the NaCl intake but CNS intake decreased the levels significantly in this study. These results indicate that the anti-obesity effects of CNS is exerted by regulating adipo/lipogenesis.

The primary degradation of fatty acids is initiated by fatty acid β-oxidation in the mitochondria^50^, and CPT-1 catalyzes one of the rate-limiting steps in β-oxidation^50^. Lipolysis of triacylglycerol stores in white adipose tissue results in the conversion of glycerol and fatty acids^51^, and HSL is known to hydrolyze TG, diglycerides and cholesteryl esters^52^. In this study, the CNS intake enhanced CPT-1 and HSL, indicating that CNS also reduces obesity by increasing the β-oxidation and lipolysis. However, NaCl intake decreased the expression of CPT-1 and HSL in liver, thereby clarifying the relationship between weight gain and NaCl in mice. Moreover, all mice were administered 45% high fat diet or HFD + different salts diet, but the lipid concentrations in feces were similar in all groups (Supplement Fig. 5). Therefore, the anti-obesity effect of CNS is regulated by adipo/lipogenesis, β-oxidation and lipolysis, and the effects of NaCl on obesity are controlled specifically by β-oxidation and lipolysis in cells but are not involved in lipid excretion. One possible reason is that CNS may regulate thermogenesis in brown adipose tissue^53^. Unfortunately, brown adipose tissue was not examined because this study focused on finding the salts and gene regulation in hepatic and white adipose tissue. Despite this, further studies on the relationship between salt intake and thermogenesis regulation are needed. Thus far, few studies have examined the relationship between salt intake and obesity genes. Therefore, these results will form the basis for future studies of salt intake and obesity related genes.

Obesity results in an increase in the relative abundance of Firmicutes but decrease Bacteriodetes^54^. However, considering the relative abundance, obesity increases Actinobacteria with a significant decrease in Bacteriodetes, and no significant difference of Firmicutes^55^. In this study, GS intake decreased Firmicutes but CNS intake increased Firmicutes at the phylum level (Supplement Fig. 8A). Therefore, the relationship between obesity and intestinal microbiota is unclear at the phylum level, and more research will be needed on the relationship of salt intake with respect to the genus of gut microbiota. High-salt (NaCl) diet increases Firmicutes but decreases Bacteroidetes in mice^56^. NaCl intake enhanced Firmicutes but reduced Bacteroidetes, similar to a previous study. On the other hand, CNS intake significantly increased Bacilli and *Atopostipes* at the genus level, which differed from the populations observed with GS intake (Supplement Fig. 8B). Hence, CNS intake affected the microbiota differently than the NaCl intake, and these results were attributed to the difference in mineral composition between GS and CNS.

As mentioned earlier, CNS has higher Na^+^ content than GS. However, the content ratio in GS was found to be much higher than in CNS, including Mg^2+^ and SO_4_2^−^. These mineralogical differences are due to manufacturing processing distinction between GS and CNS. Interestingly, the crystal structure of halite (NaCl) mineral from the GS and CNS also reveal different patterns. Analysis of the crystal plane confirms that the control and GS have an almost identical crystal structure for halite (NaCl), whereas the CNS shows a much higher peak at 45.54°(1.99Å) of (2,2,0) crystal plane. These results indicate that salt crystals in CNS are better developed.

A previous mineral analysis confirmed that the presence of more sulfate minerals than chloride minerals, especially MgSO_4_ minerals. According to the sequential precipitation of seawater analysis, since the ionic affinity between Mg^2+^ and SO_4_^2−^ is higher than Cl^−^, thus after the sulfate mineral is precipitated, the residual Mg^2+^ reacts with the Cl^−^ and precipitates as MgCl_2_^24^. However, our study samples confirm that the concentration of Mg did not result in the formation of chloride minerals. These differences are more evident in direct observations using SEM microscopic analysis and EDS spectroscopy approaches. Compared to aggregates of well-developed cubic structure of halite in the Control, GS was identified by the small size of halite crystals with same size of magnesium sulfate minerals, and CNS contained relatively larger size of halite crystals with no other identifiable sulfide minerals. Thus, these differences also indicate that the crystallinity of minerals is highly affected by the type of seawater.

Numerous human studies have reported that decreasing the Mg intake and reduced Mg concentration in the serum enhances obesity and metabolic syndrome, but increasing the Mg intake and Mg concentration in serum suppresses the same^19–25,57,58^. In addition, within six hours, the standard concentration of Mg ions ranges from 40 to 80 mmol in bowel movement^59^. Thus, an excess of Mg intake would have gastrointestinal side effects and result in magnesium toxicity. Little research has been done on the mechanisms by which Mg controls obesity. This study showed that CNS had better anti-obesity effects than GS, but CNS also had a lower Mg content than GS. Similar to previous results, 0.165 mM and 0.33 mM markedly reduced the lipid droplet, but the other concentrations produced similar effects or increased the lipid droplet compared to the Control. These concentrations were determined by reference to the magnesium content in CNS. Therefore, an appropriate Mg concentration would result in a subsequent decrease in obesity in 3T3-L1 adipocytes. Ford *et al*.^58^ reported that an increase in Mg intake reduces metabolic syndrome, but more than 466 mg/d (man) and 337 mg/d (women) Mg intake groups (BMI: 26.3) produced an increase in BMI compared to the 377–465 mg/d (man) and 264–366 mg/d (women) groups (BMI: 26.1). Therefore, enhancing the Mg intake reduces the obesity parameters, but ingesting more than the appropriate amount of Mg may increase obesity. In addition, an appropriate concentration of Mg reduces the lipogenesis related genes of SREBP-1, C/EBPα, GPAT2, DGAT1, and DGAT2 in 3T3-L1 adipocytes. Based on these results, additional studies will be needed on adequate Mg intake in humans and the relationship between Mg and obesity related mechanism.

In the same population, overweight and obesity resulted in elevated serum ALT activity^60^. In the current study, CNS intake reduced body weight and ALT activity in mice serum. Based on our results, MgCl_2_ but not NaCl exhibited ALT inhibitory activity, indicating that the active component of these two salts is Mg and not Cl or Na. Recent studies have reported hesperidin^61^, hyperoside, and β-sitosterol^62^ as compounds that inhibit the activity of the ALT enzyme. However, there is little research on the relationship between minerals and ALT enzymes. Our data indicates that MgCl_2_ binds to the active site of the ALT enzyme with a low inhibition constant (*K*_*i*_ = 1.71 mM) and has a competitive relationship with ALT substrate (L-alanine), suggesting that it may act as a preventive or therapeutic mineral for obesity. Therefore, it is believed that SS intake regulates the ALT enzyme activity, and among the various minerals contained in the SS, Mg regulates the lipid metabolism as well as liver enzyme activity. Nevertheless, more research will be needed to reveal the relationship between minerals and liver enzymes.

In conclusion, CNS suppresses obesity by regulating the mice body weight, lipid accumulation in liver and epididymal white adipose tissue, adipo/lipogenesis, β-oxidation, lipolysis in high fat diet induced obese mice and 3T3-L1 adipocytes. CNS has a different mineral composition, crystallization, and morphology compared to NaCl and GS. In particular, CNS has lower Mg and S contents than GS. Moreover, an appropriate concentration of MgCl_2_ significantly decreased obesity in 3T3-L1 adipoctyes, as well as the ALT enzyme activity. Therefore, modification of the method of SS production alters the mineral composition, which ameliorates obesity. In addition, among the various minerals present in SS, an appropriate concentration of Mg is effective on the inhibition of lipid metabolism and liver enzyme activity. Thus, instead of purified salt (NaCl, table salt), the consumption of SS with an appropriate concentration of Mg can help inhibition of obesity.

## Methods

### Reagents and sea salts

NaCl (Sigma-Aldrich Co., St. Louis, MO, USA), MgCl_2_ (Sigma-Aldrich Co.), azoxymethane (A, Sigma-Aldrich Co.), isobutylmethylxanthine (Sigma-Aldrich Co.), dexamethasone (Sigma-Aldrich Co.), insulin (Welgene Inc. Gyeongsangbuk-do, Korea), and dextran sodium sulfate (D, Dextran Sulfate Sodium Salt Reagent grade, M.W. 36,000–50,000, MP Biomedicals, LLC, France) were purchased. Various sea salts (GS, FS, CNS) were prepared by Shin-il Saltern Corporation (Shinan, Jeollanam-do, Korea). More details of the manufacturing methods of sea salts are provided in the supplement methods.

### Animal studies

Male mice (C57BL/6, six-weeks-old) were purchased from Orient Bio (Sungnam, Gyunggi-do, South Korea). The mice were housed in a specific pathogen-free (SPF) room (Cha Bio complex, Sungnam-si, Gyunggi-do, South Korea) and given access to a diet (DooYeol Biotech, Seoul, South Korea) and water *ad libitum*. The diet consisted of AIN-93G (Normal), 45% high fat diet (HFD) or HFD containing 1% of the respective NaCl and sea salt samples (Supplement Table 1). This study of the mice was used in accordance with the guidelines of the Cha Laboratory Animal Research Center, and the protocol was approved by the Institutional Animal Care and Use Committee in Cha University (IACUC-160057 and IACUC-170007). More detailed information is provided in the supplement methods.

### Histological observation and immunohistochemistry

Samples of the distal colon, liver, and epididymal fat from each animal underwent a histological examination and immunohistochemical examination. These organs were fixed in 10% (v/v) neutral-buffered formalin, dehydrated in ethanol, and embedded in paraffin. The sections were sliced to a 5-μm thickness for each sample. Hematoxylin and eosin (H&E) staining and immunohistochemistry were performed using the standard protocol and used CD56 antibody (M7304, DAKO, Carpinteria, CA, USA), HRP-conjugated monoclonal secondary goat anti-mouse IgG (K4006, DAKO) and a horseradish peroxidase-diaminobenzidine (HRP-DAB) staining kit (SK-4100, VECTOR Lab., Burlingame, CA, USA). More detail information is provided in the supplement methods.

### Serum lipid, leptin, and liver enzyme profiles analysis

Triglyceride (TG) and total cholesterol (TC) levels were measured using the respective commercially available assay kits (ASAN Pharmaceutical., Seoul, Korea). Low density lipoprotein cholesterol (LDL) was calculated using the Friedewald Equation^63^. Leptin was measured using an assay kit (BD Science, Franklin Lakes, NJ, USA), and aspartate transaminase (AST) and alanine transaminase (ALT) levels were checked using their respective assay kits (ASAN Pharmaceutical.). All procedures were performed according to the manufacturer’s suggested protocols.

### 3T3-L1 adipocytes differentiation and lipid droplet analysis

The 3T3-L1 adipocytes were purchased from the Korean Cell Line Bank (Seoul, Korea). Differentiated adipocytes were converted using isobutylmethylxanthine, dexamethasone, and insulin. Oil red O staining was used for lipid droplet analysis. More detail information is provided in the supplement methods.

### Reverse transcription polymerase chain reaction (RT-PCR) and real-time quantitative PCR (RT-qPCR) assay

The mRNA expression levels of cancer and obesity related genes in colon, liver, and epididymal tissues from mice as well as 3T3-L1 adipocytes were measured by RT-PCR and RT-qPCR assay. RNA was extracted using Trizol reagent (Invitrogen, Carlsbad, CA, USA). cDNA was obtained using oligo dT_18_ (Invitrogen), reverse transcriptase buffer (Invitrogen), dNTPs (Invitrogen), reverse transcriptase (Invitrogen), and RNase inhibitor (Invitrogen). RT-PCR was then carried out using an automatic thermocycler. Glyceraldehyde-3-phosphate dehydrogenase (GAPDH) was used as a housekeeping gene. Gene expression was quantified using ImageJ software (http://rsbweb.nih.gov/ij/). In RT-qPCR, the prepared cDNA was amplified by incorporating each primer, SYBR green (Solis biodyne, Tartu, Estonia) and cDNA using a thermal cycler BioRad CFX-96 real time system. 18s rRNA was used as a housekeeping gene. All gene primer sequences are presented in Supplement Table 2. More details are provided in the supplement methods.

### Western blotting

Protein was isolated from the liver, epididymal white adipose tissue, and 3T3-L1 cells using RIPA buffer (Invitrogen). Loading was performed using the mini-protein TGXgel (Bio-rad), and transfer was carried out using the iBlot^TM^ gel transfer system and gel transfer stack PVDF (Invitrogen). The membranes were then captured using the primary antibodies. After washing, the membranes were then captured using the respective secondary antibody. After washing, ECL (Invitrogen) was treated and detected using the LAS-4000 (GE Healthcare Bio-Sciences AB, Björkgatan, Uppsala, Sweden). Gene expression was quantified using ImageJ software. Membranes were then captured using the primary antibodies SREBP-1 (sc-365513, Lot# D1513), C/EBPα (sc-365318, Lot# H0613), FAS (sc-1024, Lot# G0312), GPAT2 (sc-168448, Lot# A1811), DGAT1 (sc-26173, Lot# C2315), DGAT2 (sc-66859, Lot# A1215), CPT-1 (sc-139482, Lot# H2213), Actin (sc-8432, Lot# C3017) (Santa Cruz Biotechnology Inc., Dallas, TX, USA), and the respective secondary antibody (Santa Cruz Biotechnology Inc.). More detail information is provided in the supplement methods.

## Characteristics of Sea Salts

### Inductively coupled plasma optical emission spectrometry (ICP-OES)

The metal cation mineral contents of solar salts were analyzed by ICP-OES (Optima 8300, PerkinElmer, Waltham, MA, USA) under the following conditions: operating power, 1.5 kW; Nebulizer, mira mist, plasma gas flow, 12.0 L/min; auxiliary gas flow, 0.2 L/min; nebulizer gas flow, 0.55 L/min. The detection limit for the cations was determined to be 10 ppb.

### Ion chromatography

Dissolved samples (NaCl, GS, and CNS) were analyzed by ion chromatography (IC, Dionex ICS-5000+ Reagent-Free HPIC System, Thermo Inc., USA) for anion analysis (including chloride and sulfate), which have a detection limit of 1 ppm for Cl^−^ and SO_4_2^−^.

### X-ray diffractometer (XRD)

XRD was performed on air-dried samples (NaCl of control, GS, and CNS) at Yonsei University using an Ultima IV (Rigaku, Japan) model automated X-ray diffractometer with Cu-Kα radiation. Oriented powder mount samples were placed on the holder. XRD profiles, over ranges of 2θ degrees, ranging from 3° to 100°, were recorded at scan speeds of 1.5°/min and step sizes of 0.02° with a receiving slit size of 0.3 mm and a divergence slit size of 1.25°. Crystallographica Search-Match software (v.2.0.3.1) was used to identify the composition of minerals constituting each solar salt sample.

### Scanning electron microscopy (SEM)

The inherent mineral morphology and texture were observed on secondary electron images (SEI) using field emission SEM (JEOL-7610F-Plus, JEOL, Japan) equipped with EDS operating at 15 KeV with a working distance of 8 mm; these experiments were performed at Yonsei University. Briefly, fine grains of all three samples (GS, CNS, and Control) were placed on a carbon tape and coated with platinum.

### ALT enzyme activity and enzyme kinetic analysis

The ALT activity inhibitory effect of MgCl_2_ (Sigma) and NaCl (Sigma) was carried out using the alanine transaminase colorimetric activity assay kit (Cayman Chemical, Ann Arbor, MI, USA) according to the manufacture’s instruction with the selected modifications. Kinetic models, Dixon plot, and Lineweaver-Burk plot were used to determine the mechanism of ALT inhibition. More detail information is provided in the supplement methods.

### Statistical analysis

Data are presented as the mean ± standard deviation (SD), and RT-qPCR data are presented as the mean ± standard error (SE). Differences between the mean values for individual groups were assessed using one-way analysis of variance (ANOVA) of Duncan’s multiple range tests. Differences were considered significant when *P* < 0.05. The SPSS v18 statistical software package (SPSS Inc. Westlands, Hong Kong) was used to check these analyses.

## Supplementary information

Dataset 1.

## Data Availability

All data generated or analyzed during this study are included in this published article (and its Supplementary Information Files).
