# Supplementary material for: Cube natural sea salt ameliorates obesity in high fat diet-induced obese mice and 3T3-L1 adipocytes
Source: Sci Rep. 2020 Feb 25;10:3407. doi: 10.1038/s41598-020-60462-z (PMC7042290; doi:10.1038/s41598-020-60462-z)
Supplement: Supplementary file 1 — Dataset 1. [file 41598_2020_60462_MOESM1_ESM.docx]

**Cube natural sea salt ameliorates obesity in high fat diet-induced obese mice and 3T3-L1 adipocytes**

Eui-Seong Park, Yu Ting, Kiho Yang, Shinil Choi, Seung-Min Lee, Kun-Young Park

Inventory of Supplemental Information

　　Supplemental methods

Supplemental result

Supplemental full-length gels and blots

**Supplement methods**

**Manufacturing methods of the sea salt**

The production process for sea salts was as follows: seawater was blown in a reservoir (salty water concentration: 1~2℃) and then transferred to an evaporating salt field (salty water concentration: 2~8℃). Seawater was then transferred to a second evaporating salt field (salty water concentration: 9~17℃) and then to a crystallizing salt field (salty water concentration: 25℃). In the crystallizing salt field, seawater was a cohesion of crystals (salty water concentration: 27~28℃). Then, salt crystals were collected (salty water concentration: 27~28℃)^1^. Generally, when producing sea salts, new seawater (50±5%) is mixed with old remaining seawater (50±5%) after collection of sea salts. Therefore, we prepared three types of sea salts. Conventionally manufactured sea salt was a mixture of concentrated old (50±5%) and new seawater (50±5%) during evaporation in the wind and sunlight (GS). A mixture of concentrated old (50±5%) and new seawater (50±5%) was filtered through a charcoal and magnetic filter and then evaporated using the same method as GS (FS). sea salt was made using only concentrated new seawater (100%) during evaporation in the wind and sunlight (CNS)^2^.

**Animal studies**

Mouse diets consisted of AIN-93G (3800 kcal) and 45% high fat diet (4600 kcal) (55, 56), and the sea salt samples (1%) altered the high fat diet composition (4554 kcal) (Supplement table 1). The combination of 1% salts in the diet was appropriate according to our previous studies^3,4^.

A/D+HFD study : The animals were randomly divided into six groups of 10 mice each, and diets were as follows. Normal: no treatment and AIN-93G diet; HFD: 45% high fat diet; A/D+HFD: HFD+AOM/DSS; A/D+HFD+GS: HFD+AOM/DSS+GS; A/D+HFD+FS: AOM/DSS+HFD+FS; A/D+HFD+CNS: AOM/DSS+HFD+CNS. AOM/DSS treatment groups were given a single intraperitoneal injection of AOM (10 mg/kg, AOM, Sigma Co., St. Louis, MO, USA). Animals received 2.5% DSS (Dextran Sulfate Sodium Salt Reagent grade, M.W. 36,000-50,000, MP Biomedicals, LLC, France) in the drinking water for 7 days at 1 and 3 weeks. All mice were sacrificed at the end of the experiment (8 weeks) and were fasted for 12 h, except the HFD group. Colon tissue was collected for analysis of colon length and tumor quantification, and liver and fat tissues were also collected and stored in a −80℃ refrigerator for gene expression analysis.

HFD study : The animals were randomly divided into four diet groups of 10 mice each as follows: HFD group, 45% high fat diet; NaCl group, HFD+NaCl (1%, Sigma-Aldrich Co., St. Louis, MO, USA); GS group, HFD+GS (1%); CNS group, HFD+CNS (1%). Treatment was administered to all groups for 17 weeks. All mice were sacrificed at 17 weeks. Serum, liver and epididymal white adipose tissue were collected and stored at −80℃ for obesity evaluating the basic parameters and for various genes expression analysis.

Mice weights and dietary weights were measured weekly using the same balance at the same time. Mice average food intake per week (AFI) and feed conversion ratio (FCR) were calculated by the following formula^5^. We adjusted the original formula by adding 5 to the denominator since the FCR value becomes abnormal if weight loss or gain is too small, which results in a decimal or negative value in the denominator.

Average food intake in a week/mice (AFI) (g) = $\frac{(Total food intake in a week)(g)}{(Mice number)}$,

Feed conversion ratio (FCR) =$\frac{(Feed consumption in a week)(g)}{(Mouse body weight gain in a week+5)(g)}$

**Histological observation and immunohistochemistry**

**H&E staining :** The sections were deparaffinized in xylene and rehydrated through a graded ethanol series. Hematoxylin and eosin (H&E) staining was performed according to the standard protocol^6^. Images were acquired using a Nikon Microscope ECLIPSE 50i (Nikon Inc., Tokyo, Japan) equipped with an infinity camera (SPOT RT741 Slider Color, Diagnosis Instruments, MI, USA).

**3T3-L1 adipocytes differentiation method and lipid droplet analysis**

Pre-adipocyte state 3T3-L1 cells were cultured in Dulbecco’s Modified Eagle’s Medium (DMEM, Sigma) supplemented with 10% bovine calf serum (BCS, Welgene Inc., Gyeongsangbuk-do, Korea) and 1% penicillin-streptomycin (PS, Welgene Inc.). Pre-adipocytes were plated on a 6-well plate at a density of 2 × 10^5^ cells/mL; cell density was counted and adjusted using a cell counter (Luna automated cell counter, Logos Biosystems, Gyeonggi-do, Korea). The culture was incubated for 48 hours. The pre-adipocyte medium was then changed to DMEM with 10% fetal bovine serum (FBS, Sigma) and 1% PS in a mixture of 0.5 mM isobutylmethylxanthine (Sigma), 0.25 μM dexamethasone (Sigma), and 1 μg/mL of insulin (Welgene Inc.). Cell cultures were further incubated for 48 hours to convert differentiated adipocytes. Cells were then cultured for 6 days in DMEM, 10% FBS, 1% PS, and 1 μg/mL of insulin medium to prepare differentiated adipocytes. Different concentrations of MgCl_2_ (Sigma) were mixed with the medium and cultured for 24 hours in the well-differentiated adipocytes in a 6-well plate.

Following termination of the experiment, the medium was discarded and cells were fixed with 10% formalin. After fixation, the cells were reacted with Oil Red O working solution (Sigma), washed with distilled water, and observed under a microscope (U-Tvix-2, Olympus, Tokyo, Japan). Isopropanol mixed with Oil red O absorbance was measured at 490 nm with a spectrophotometer (Wallac Victor3 1420 Multilabel Counter, PerkinElmer, Waltham, MA, USA).

**Reverse transcription polymerase chain reaction (RT-PCR) and real-time quantitative PCR (RT-qPCR) assay**

Total RNA was isolated from colon and liver tissues in mice and adipocytes using Trizol reagent (Invitrogen, Carlsbad, CA, USA) and centrifuged at 12,000 × g for 10 min at 4℃ after addition of chloroform. Isopropanol was added to the supernatant at a 1: 1 ratio, and RNA was pelleted by centrifugation (12,000 × g for 10 min at 4℃). After washing the pellet with 75% ethanol, RNA was resolved in diethyl pyrocarbonate (DEPC)-treated RNase-free water and quantified by measuring the absorbance at NanoDrop ND-1000 (NanoDrop Technologies Inc., Wilmington, DE). Equal amounts of RNA (2 µg) were attached to 500 ng of oligo dT_18_ (Invitrogen) primers and reverse transcribed in a master mix (Invitrogen) containing 1 × reverse transcriptase buffer, 1 mM dNTPs, 140 U of MMLV reverse transcriptase, and 40 U of RNase inhibitor for 120 min at 37℃ and 2 min at 95℃. In RT-PCR, PCR was then carried out using an automatic thermocycler (Bioneer, Daejeon, South Korea). The PCR products were divided in 2% agarose gels and visualized by EtBr staining. Glyceraldehyde-3-phosphate dehydrogenase (GAPDH) was used as a housekeeping gene. Gene expression was quantified using ImageJ software (<http://rsbweb.nih.gov/ij/>). In RT-qPCR, the prepared cDNA was amplified by incorporating each primer, SYBR green (Solis biodyne, Tartu, Estonia) and cDNA using a thermal cycler BioRad CFX-96 real time system (BioRad, Hercules, CA, USA) (95°C 15 min → 50 cycle (95°C 30 sec → 65°C 30 sec → plate read)), after which the expressed genes were analyzed. All gene primer sequences are presented in Supplement Table 2.

**Western blotting**

Protein content was estimated by the Bradford assay (Bio-rad, Hercules, CA, USA). Loading was done using mini-protein TGXgel (Bio-rad), and transfer was carried out using the iBlot^TM^ gel transfer system and gel transfer stacks PVDF (Invitrogen). Membranes were blocked in 5% skim milk, and then washed with PBS-T and PBS. Membranes were then captured using the primary antibodies SREBP-1 (sc-365513, Lot# D1513), C/EBPα (sc-365318, Lot# H0613), FAS (sc-1024, Lot# G0312), GPAT2 (sc-168448, Lot# A1811), DGAT1 (sc-26173, Lot# C2315), DGAT2 (sc-66859, Lot# A1215), CPT-1 (sc-139482, Lot# H2213), and Actin (sc-8432, Lot# C3017) (Santa Cruz Biotechnology Inc., Dallas, TX, USA) overnight. After washing, the respective secondary antibody (Santa Cruz Biotechnology Inc.) was diluted appropriately in 2.5% skim milk and the probed membranes were subjected to reaction. After washing, ECL (Invitrogen) was treated and detected using the LAS-4000 (GE Healthcare Bio-Sciences AB, Björkgatan, Uppsala, Sweden). After detection, membranes were washed and stripped (stripping buffer, Invitrogen) and the previous step was again repeated. Gene expression was quantified using the ImageJ software.

**ALT enzyme analysis**

The ALT activity inhibitory effect of MgCl_2_ (Sigma) and NaCl (Sigma) was carried out using the alanine transaminase colorimetric activity assay kit (Cayman Chemical, Ann Arbor, MI, USA) according to the manufacture’s instruction, with selected modifications. Essentially, in each well of a 96-well plate, ALT enzyme (10 μL/well) was diluted with ALT assay buffer containing 100 mM Tris-HCl, pH 7.8, 10 mM sodium bicarbonate, 0.1 mM pyridoxal-5-phosphate and 0.01% sodium azide, added with or without test sample. Crystalline L-alanine was dissolved in assay buffer to make the working solution at a 300 mM concentration; 75 μL/well was added into 96-well plate as the substrate. Subsequently, 10 μL ALT cofactor was added in each well containing a lyophilized powder of NADH and lactate dehydrogenase (LDH), dissolved in 1.5 mL ALT assay buffer. After incubation at 37°C for 15 min, the reaction was initiated by 150 mM α-ketoglutarate. The absorbance was then measured at 340 nm with a Wallac Victor3 1420 Multilabel Counter (Perkin-Elmer) immediately after 5 min incubation at 37°C. Principally, ALT activity is measured by monitoring the rate of NADH oxidation in a coupled reaction system employing LDH; the oxidation of NADH to NAD^+^ is accompanied by a decrease in absorbance at 340 nm. β-chloro-L-alanine hydrochloride (Sigma) was used as a positive control. The inhibition (%) was calculated as {(A_control_ - A_color_ - A_sample_) / A_control_} X 100 where A_control_ was the absorbance of control group, A_color_ was the absorbance of color group, and A_sample_ was the absorbance of sample group. The test sample (MgCl_2_, NaCl, and β-chloro-L-alanine hydrochloride) was expressed in terms of as IC_50_ value (μM) as calculated from the log-dose inhibition curve.

**Enzyme kinetic analysis with ALT**

ALT inhibitory effect was evaluated by monitoring the effects of different concentrations of substrates (100, 200, and 300 mM L-alanine), with or without different sample concentrations (0.5, 1, and 2 mM). The enzymatic procedure consisted of the aforementioned ALT inhibitory assay methods. Obtained data were evaluated using the SigmaPlot 12.0 software, and the inhibition constants (*K_i_*) were determined via an interpretation of the Dxion plot, where the value of the x-axis implies – *K_i_*.

**Microbiota analysis**

**MiSeq-Metagenomic sequencing from sample QC to sequencing**

DNA was extracted using the PowerSoil® DNA Isolation Kit (MO BIO Laboratories, Inc., Carlsbad, CA USA), according to the manufacturers’ protocol. Each sequenced sample was prepared using the Illumina 16S Metagenomic Sequencing Library protocols. The 16S rRNA genes were amplified using 16S V3-V4 primers (Forward: 5'-TCG TCG GCA GCG TCA GAT GTG TAT AAG AGA CAG CCT ACG GGN GGC WGC AG-3', Reverse: 5'-GTC TCG TGG GCT CGG AGA TGT GTA TAA GAG ACA GGA CTA CHV GGG TAT CTA ATC C-3').

Input gDNA was amplified with 16S V3-V4 primers, and a subsequent limited‐cycle amplification step was performed to add multiplexing indices and Illumina sequencing adapters. The final products were normalized and pooled using PicoGreen, and the size of libraries were verified using the TapeStation DNA screentape D1000 (Agilent, Santa Clara, CA, USA). This was followed by sequencing using the MiSeq™ platform (Illumina, San Diego, USA). All procedures were executed at Macrogen Inc. (Seoul, Korea).

**Operational taxonomic unit (OUT) analysis for MiSeq**

After sequencing of the MiSeq raw data, FASTQ files were made using the MiSeq Control Software (v. 2.2) and Bcl2fastq (v.1.8.4). The paired-end data separated by each sample were used to sort out only high-quality sequences (120-160 bp overlap and a length of 440-465 bp) using fast length adjustment of short reads (FLASH, v.1.2.11). High-quality sequences removed sequencing errors such as low-quality sequences, ambiguous sequences, and chimera sequences; this was achieved using the OTU analysis program (CD-HIT-OUT) based on CD-HIT-EST. Clustering among sequences with more than 97% sequence similarity yielded species-level OTU.

The representative sequence of each OTU was subjected to BALSTN (v.2.4.0) in a reference data base (NCBI 16S Microbial) to assess the taxonomic assignment of the organism information of subject having the highest similarity. If the query coverage of the best hit matching DB was less than 85% and the identity of the matched domain was less than 85%, the taxonomy was characterized as ‘not defined’.

Numerous microbial communities were analyzed using the QIIME (v.1.8) with OTU information. To confirm species diversity and homogeneity of the microbial community in the samples, the Shannon Index and Inversed Simpson Index were obtained, and alpha diversity information was confirmed through Rarefaction curve and Chao1 values. All procedures were performed at the Macrogen Inc. (Seoul, Korea).

**Cytotoxicity analysis of sea salts and NaCl in pre-adipocytes and differentiated adipocytes (MTT assay)**

Pre-adipocytes cells were plated a concentration of 1.5×10^4^ cells/mL in 96-well plates, and well differentiated 3T3-L1 adipocytes cells were plated in 12-well plates to a concentration of 1.0×10^5^ cells/mL using a cell counter (Luna automated cell counter, Logos Biosystems) and incubated for 24 hours. After incubation, the new medium was mixed with various concentrations of sea salts and NaCl and incubated for 24 hours, followed by addition of 500 μg/mL of 3-(4,5-dimethylthiazol-2-yl)-2,5-diphenyltetrazolium bromide (MTT) solution prepared with PBS and incubation for 4 hours. Then, DMEM dissolved the formazan crystals to incubate for 20 minutes in CO_2_ incubator and then measured at 550 nm using a Wallac Victor3 1420 Multilabel Counter (Perkin-Elmer, Wellesley, MA, USA).

**Supplement results**


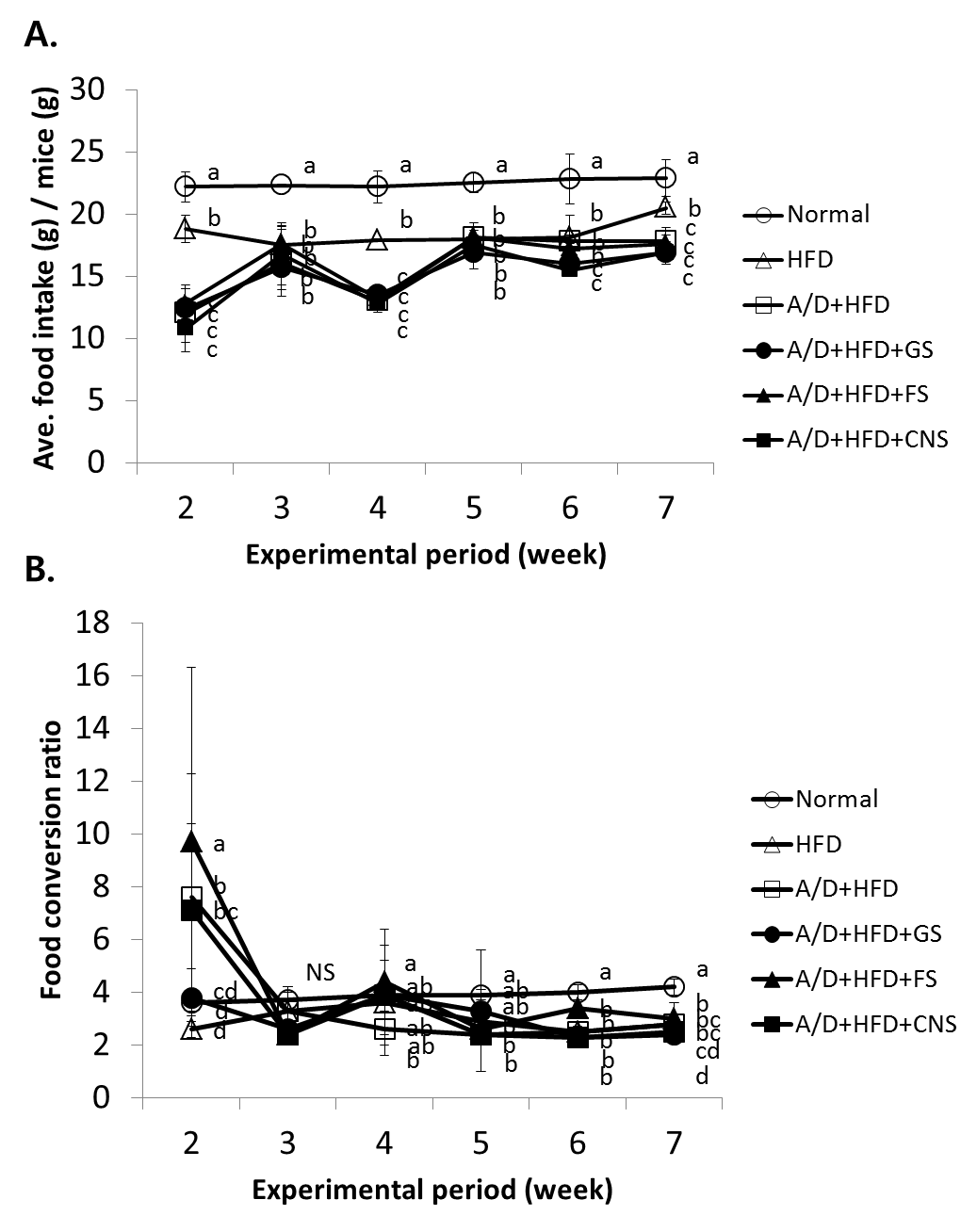


Supplement Figure 1. **(A)** Mouse average food intake/mice, and **(B)** food conversion ratio in AOM/DSS+HFD-induced colon cancer and obese mice.

Normal: AIN-93G diet, HFD: 45% high fat diet, A/D+HFD: AOM/DSS+45% high fat diet, A/D+HFD+GS: AOM/DSS+45% high fat diet + generally manufactured sea salt (mixture of concentrated old and new seawater)(1%), A/D+HFD+FS: AOM/DSS+45% high fat diet + FS: Filtering processed sea salt (mixture of concentrated old and new seawater filtered through a charcoal and magnetic filter)(1%), A/D+HFD+CNS: AOM/DSS+45% high fat diet + Cube natural sea salt (sea salt made from only concentrated new seawater)(1%)


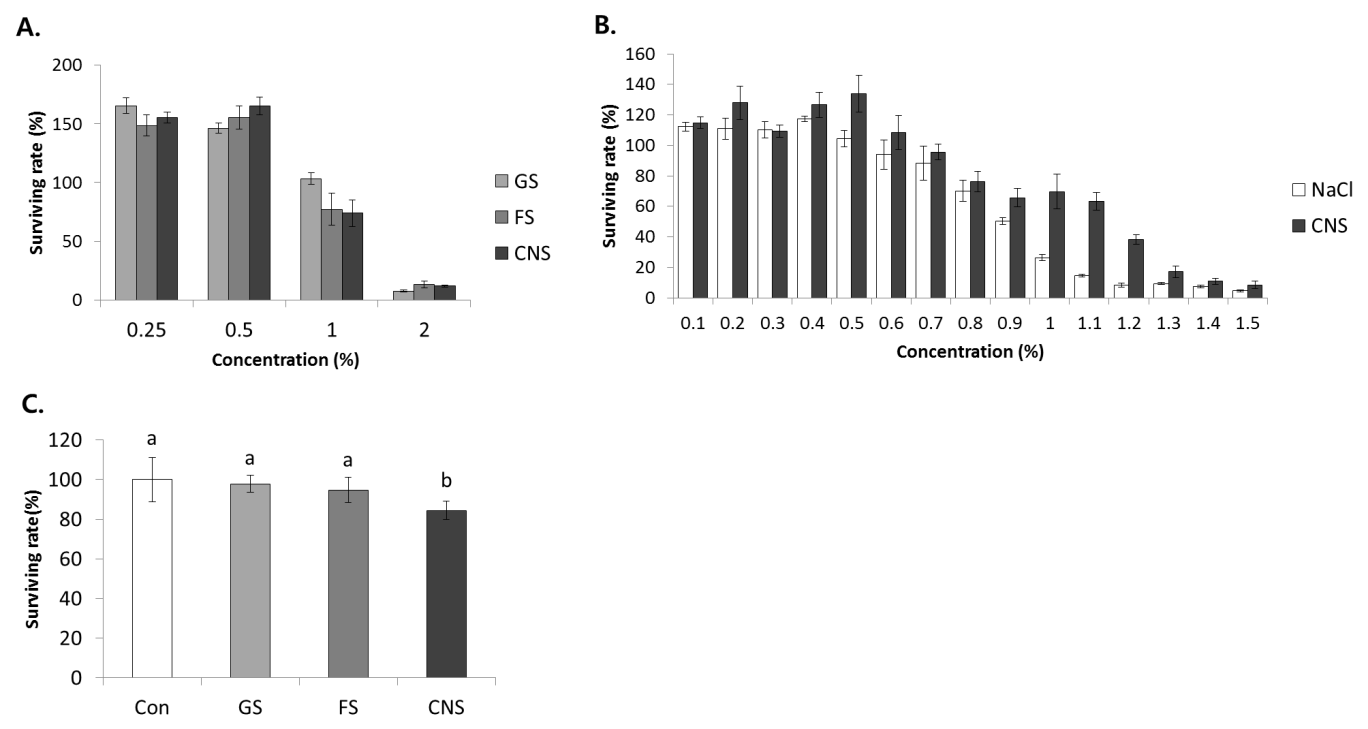


Supplement Figure 2. Cytotoxicity (MTT assay) of **(A)** different concentrations of sea salts and **(B)** NaCl in pre-adipocytes, **(C)** different sea salts (1%) in differentiated 3T3-L1 adipocytes.

GS: Generally manufactured sea salt (mixture of concentrated old and new seawater)(1%), FS: Filtering processed sea salt (mixture of concentrated old and new seawater filtered through a charcoal and magnetic filter)(1%), CNS: Cube natural sea salt (sea salt made from only concentrated new seawater)(1%).

^a-d^Means with different letters are significantly different (P<0.05) by Duncan’s multiple range test.


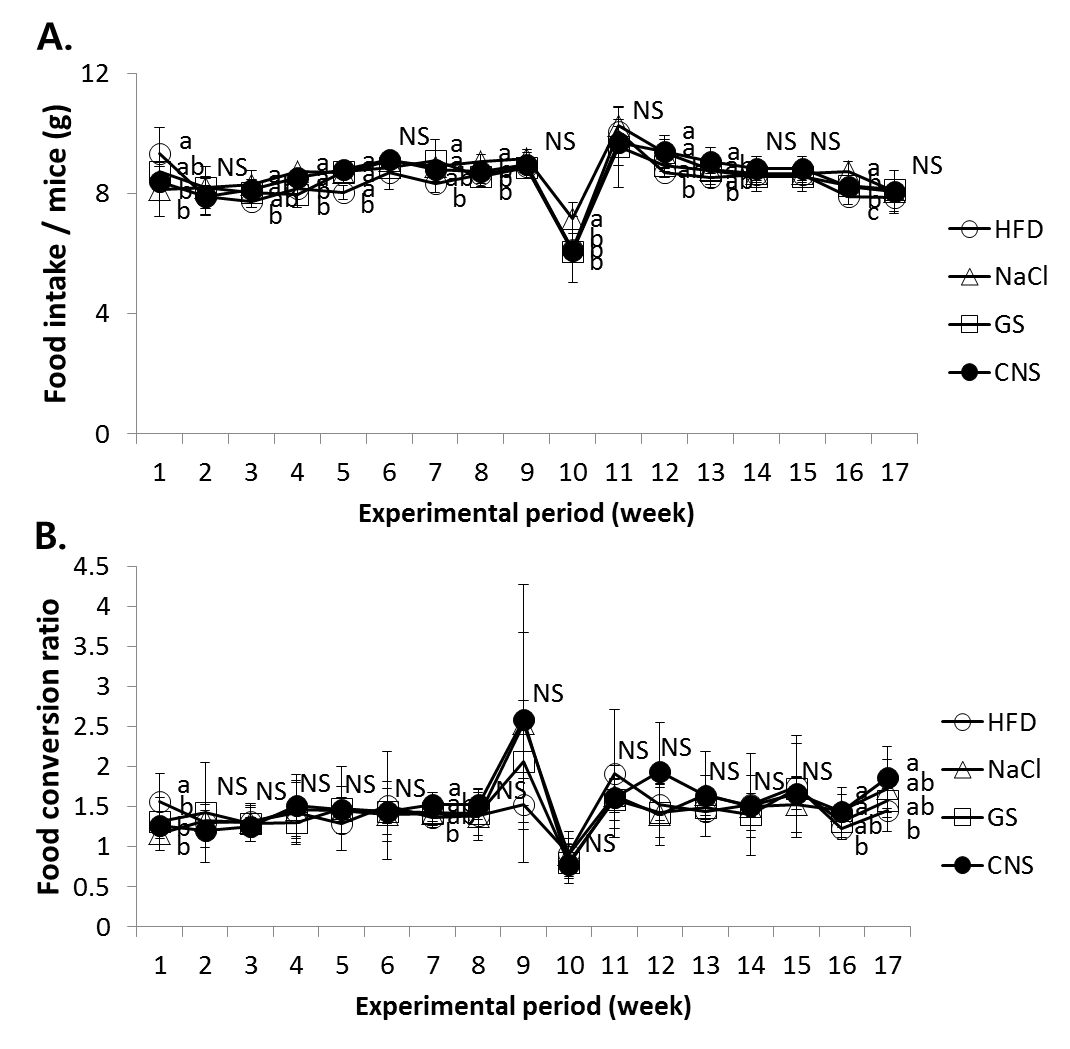


Supplement Figure 3. Food intake and food conversion ratio in high fat diet treated mice. (**A)** Average food intake (food consumption (g) / mice (g) / week). (**B)** Food conversion rate (food consumption (g) / mice body weight gain +5 (g) / week).

^a,b^Means with different letters are significantly different (*P* < 0.05) by Duncan’s multiple range test. NS: not significantly different.


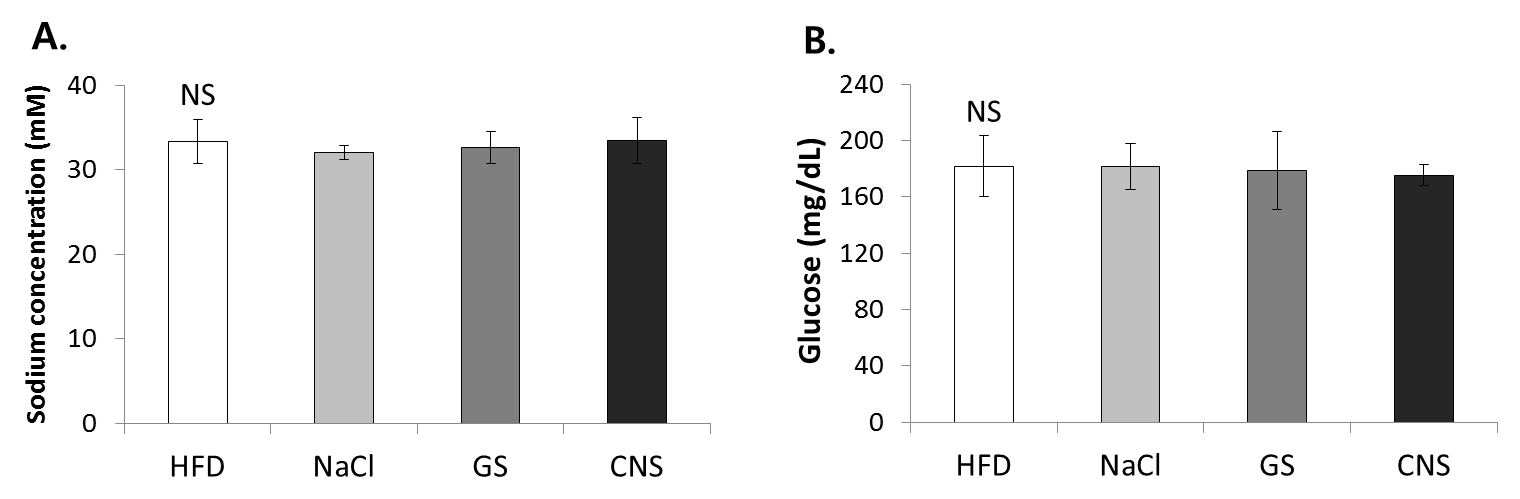


Supplement Figure 4. Glucose, insulin, and sodium levels in high fat diet induced obese mice serum. **(A)** Sodium concentration and **(B)** Glucose levels in serum at 17 weeks. All experiments used commercial kit and manufactures’ protocols.

NS: not significantly different.


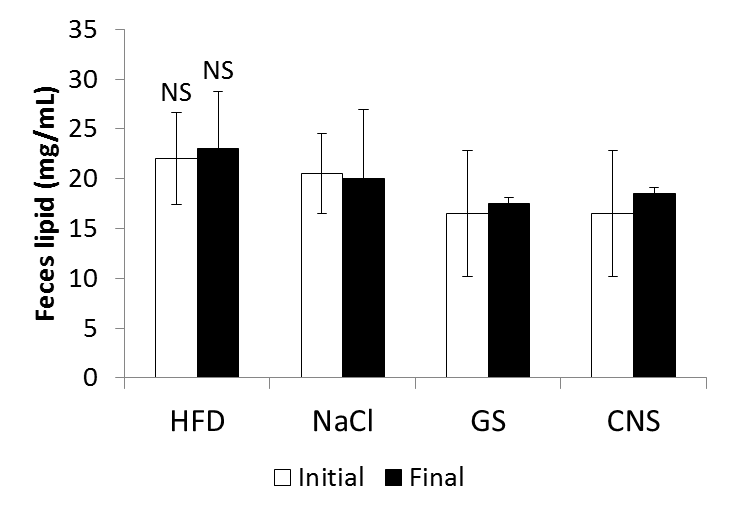


Supplement Figure 5. Lipid levels in mice feces at 17 weeks.

NS: not significantly different


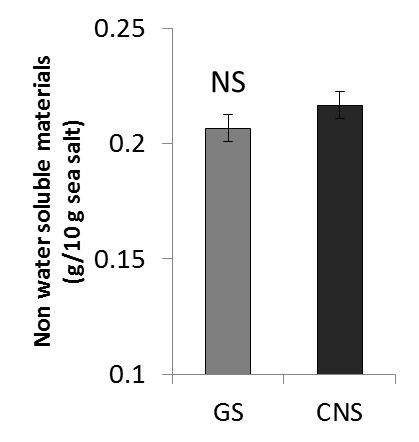


Supplement Figure 6. Non water-soluble materials in sea salts.

NS: not significantly different


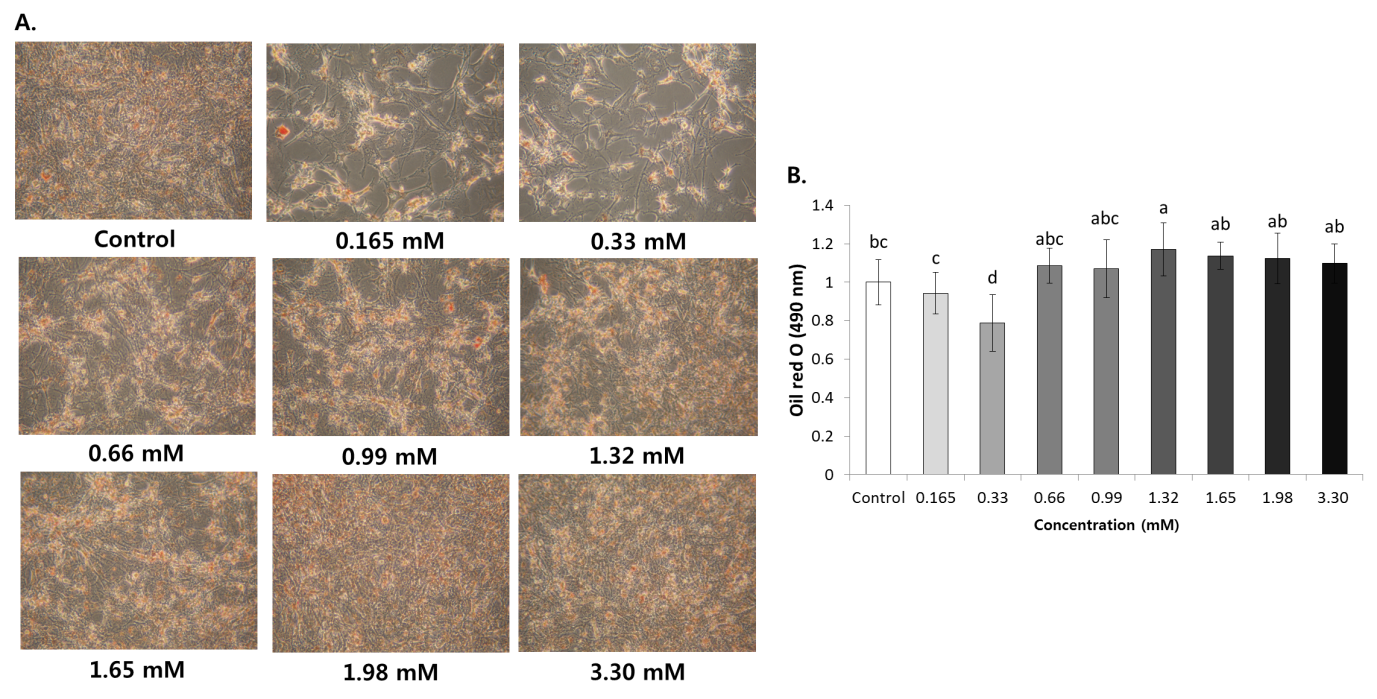


Supplement Figure 7. Appropriate concentrations of MgCl_2_ reduce lipid droplet accumulation. **(A)** Microscopy observation **(B)** Optical density (OD, 490 nm) of 3T3-L1 cells treated with different concentrations of MgCl_2_. ^a-d^Means with different letters are significantly different (*P* < 0.05) by Duncan’s multiple range test.


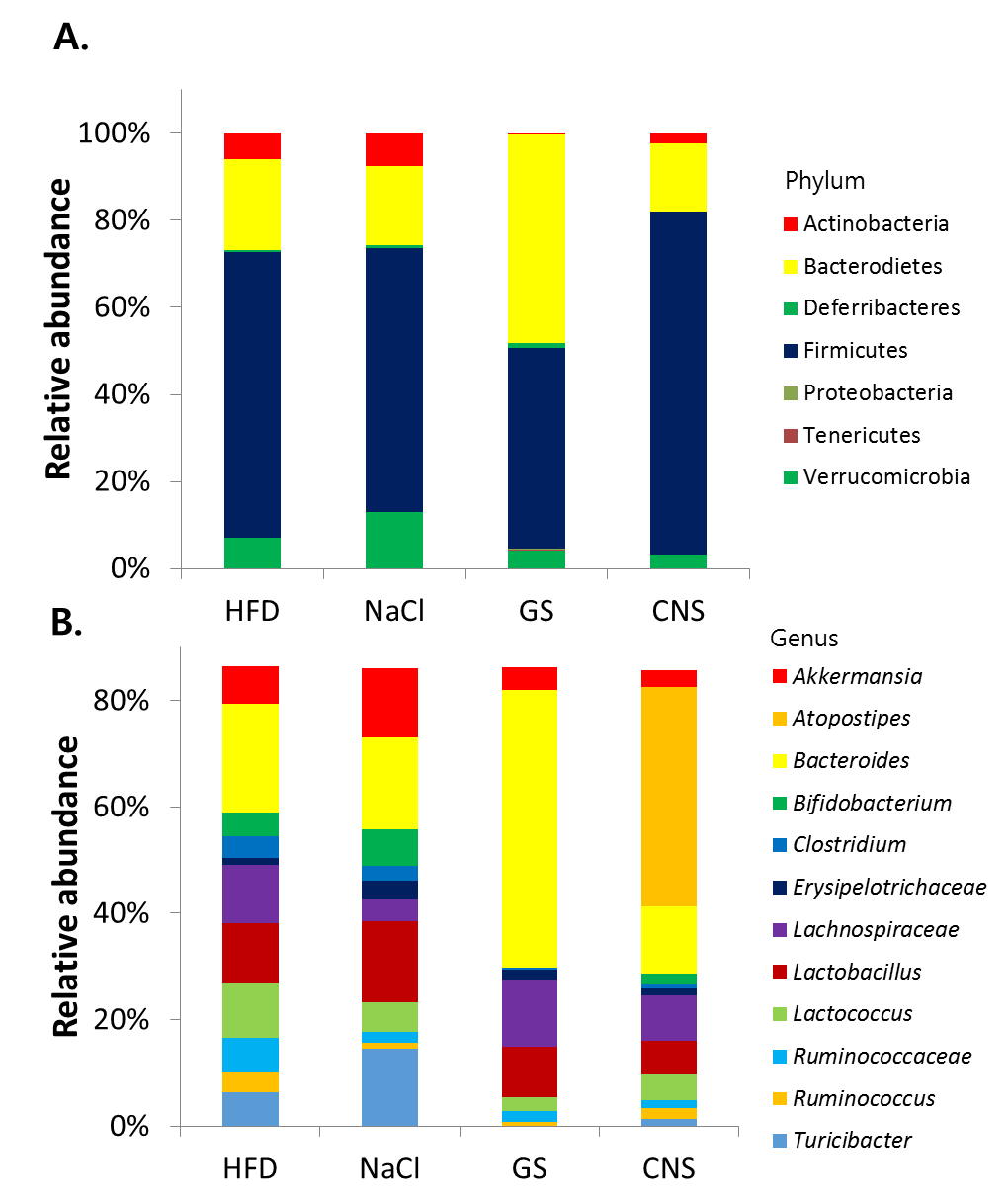


Supplement Figure 8. Different salt intake affects **(A)** phylum and **(B)** genus levels of intestinal microbiota in high fat diet induced obese mice.

Supplement Table 1. Composition of mouse diets

| Ingredient | AIN-93G | 45% HFD | 45% HFD  +sea salts |
| --- | --- | --- | --- |
| Casein | 200.0 | 245.0 | 242.55 |
| L-Cysteine | 3.0 | 3.5 | 3.465 |
| Corn Starch | 397.486 | 85.0 | 84.0 |
| Maltodextrin | 132.0 | 115.0 | 113.85 |
| Sucrose | 100.0 | 200.0 | 198.0 |
| Cellulose | 50.0 | 58.0 | 57.42 |
| Soybean Oil | 70.0 | 30.0 | 29.7 |
| Lard | - | 195.0 | 193.05 |
| AIN-93G Mineral Mix | 35.0 | 43.0 | 42.57 |
| Calcium Phosphate, dibasic | - | 3.4 | 3.366 |
| AIN-93G Vitamin Mix | 10.0 | 19.0 | 18.81 |
| Choline Bitartrate | 2.5 | 3.0 | 2.97 |
| TBHQ, antioxidant | 0.014 | - | - |
| Red Food color | - | 0.1 | 0.099 |
| 1% sea salts | - | - | 10.0 |
| Total(g) | 1000 | 1000 | 1000 |
| Kcal | 3800 | 4600 | 4554 |

Supplement Table 2. Primer sequence for real time-quantitative polymerase chain reaction

| SREBP-1 | Forward | 5’-CGG AGA CAG GGA GTT CTC AG-3’ |
| --- | --- | --- |
|  | Reverse | 5’-TGG GGG ATA TGC TCT ACC AG-3’ |
| C/EBPα | Forward | 5’-TGC TGG AGT TGA CCA TGT AC-3’ |
|  | Reverse | 5’-AAA CCA TCC TCT GGG TCT CC-3’ |
| LXRα | Forward | 5’-GCA ACT CAA TGA TGC CGA GT-3’ |
|  | Reverse | 5’-CGT GGG AAC ATC AGT CGG TC-3’ |
| PPARγ | Forward | 5’ -TTT TCA AGG GTG CCA GTT TC-3’ |
|  | Reverse | 5’-AAT CCT TGG CCC TCT GAG AT-3’ |
| FAS | Forward | 5’-TGG GTT CTA GCC AGC AGA GT-3’ |
|  | Reverse | 5’-ACC ACC AGA GAC CGT TAT GC-3’ |
| GPAT2 | Forward | 5’-ATC CTA CTG CTG CAC CT-3’ |
|  | Reverse | 5’-ACA GCA GCT TTG CAC TCA GA-3’ |
| DGAT1 | Forward | 5’-GCA GAC CGC GAG TTC TAC AG-3’ |
|  | Reverse | 5’-CTC ATG GAA GAA GGC TGA GG-3’ |
| DGAT2 | Forward | 5’-TAC AAG CAG GTG ATC TTT GA-3’ |
|  | Reverse | 5’-GGG CGA AAC CAA ATA TAC TC-3’ |
| LPL | Forward | 5’-CAG CTG GGC CTA ACT TTG AG-3’ |
|  | Reverse | 5’-CCT CTC TGC AAT CAC ACG AA-3’ |
| CPT-1 | Forward | 5’-TAT CGC CAC CTG CTG AAC C-3’ |
|  | Reverse | 5’-TTG AAG GTG ACG AAG GTG GT-3’ |
| HSL | Forward | 5’-AGA CAC CAG CCA ACG GAT AC-3’ |
|  | Reverse | 5’-CAT CAC CCT CGA AGA AGA GC-3’ |
| Bcl-2 | Forward | 5’-GAT GCT GGA GAT GCG GA-3’ |
|  | Reverse | 5’-AGA CGT CCT GGC AGC CA-3’ |
| GAPDH | Forward | 5’-CGG AGT CAA CGG ATT TGG TC-3’ |
|  | Reverse | 5’-AGC CTT CTC CAT GGT GGT GA-3’ |
| 18S rRNA | Forward | 5’-TCG AGG CCC TGT AAT TGG AA-3’ |
|  | Reverse | 5’-CCC TCC AAT GGA TCC TCG TT-3’ |

**Full-length gels and blots**


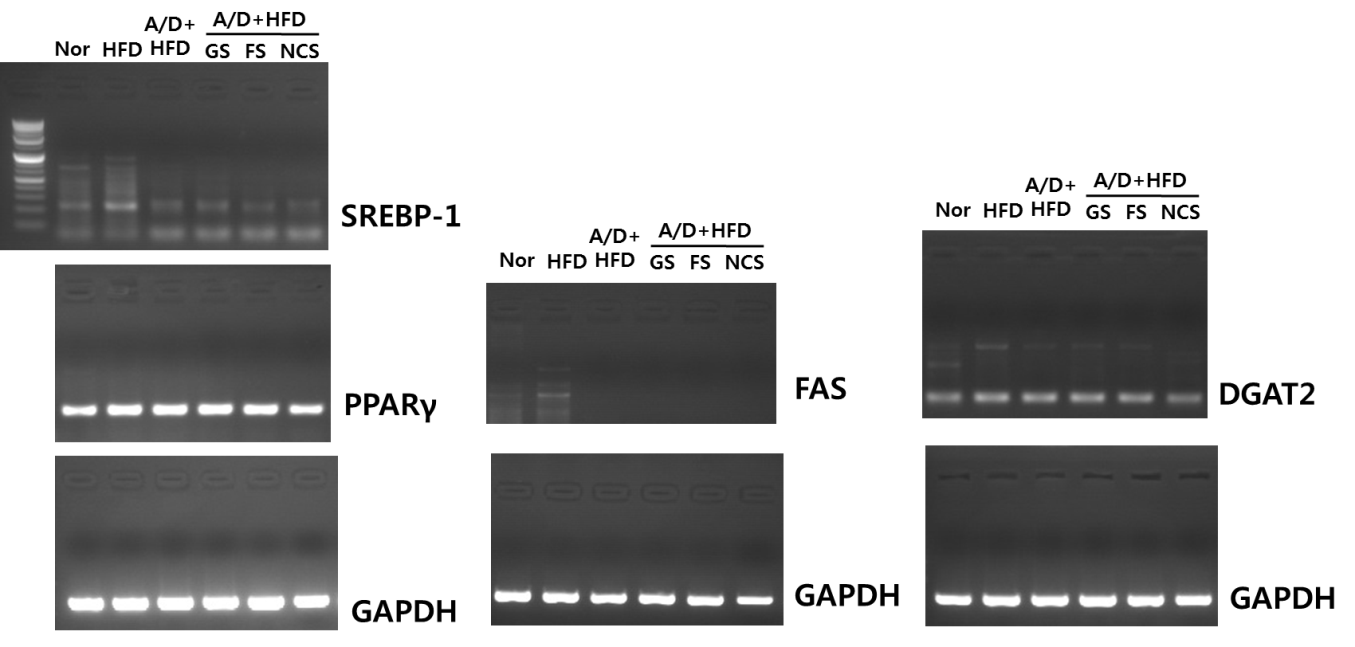


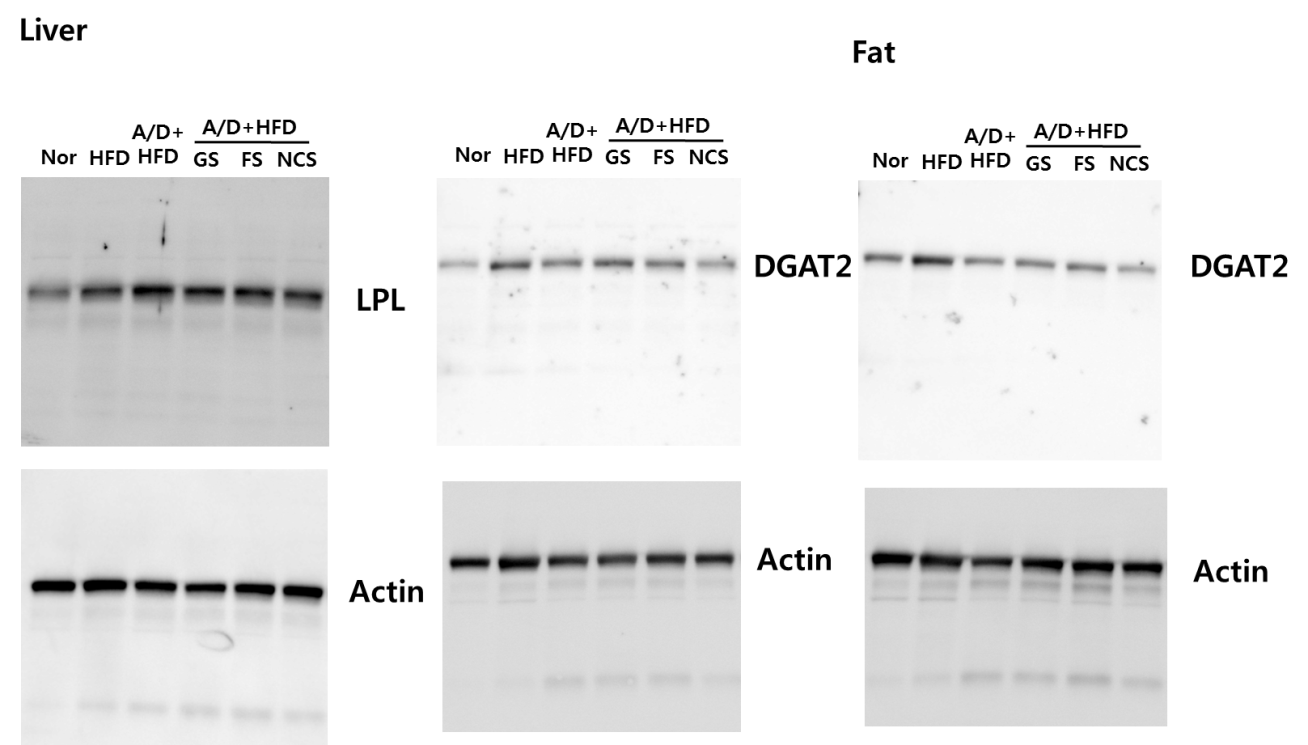


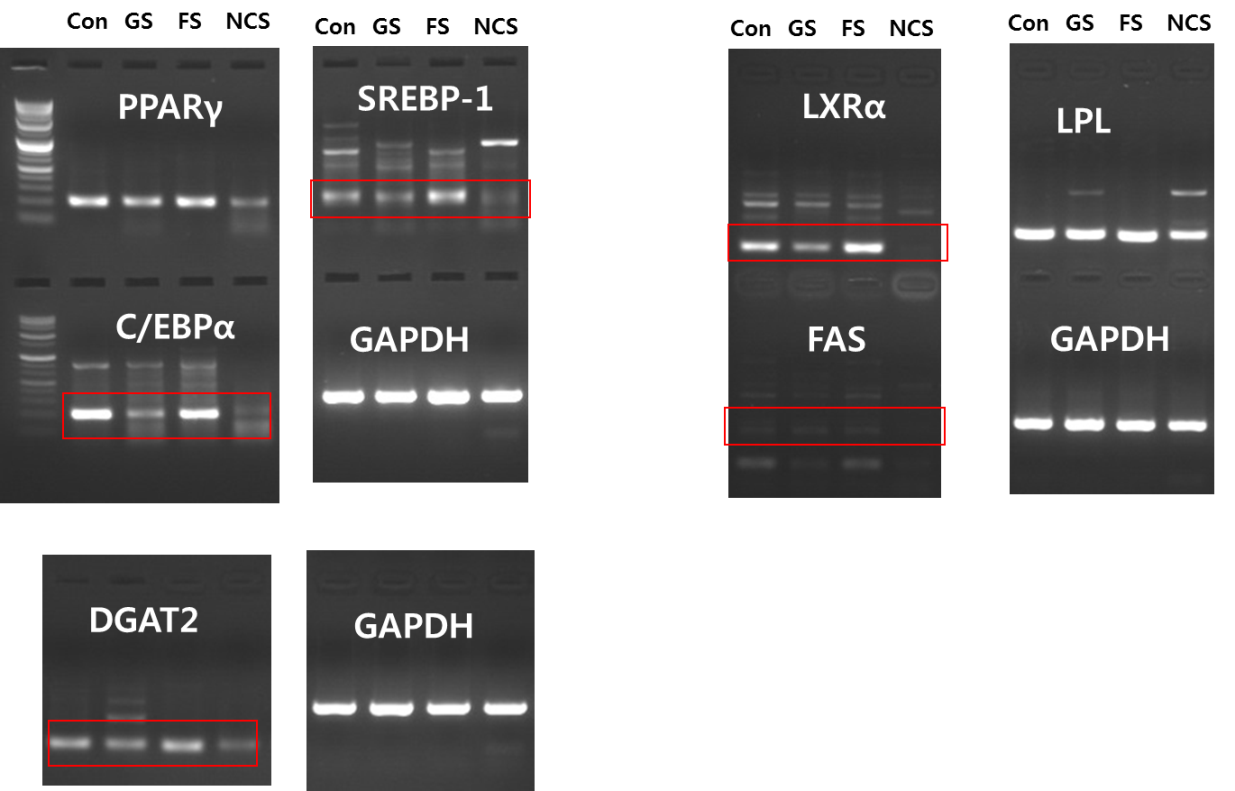


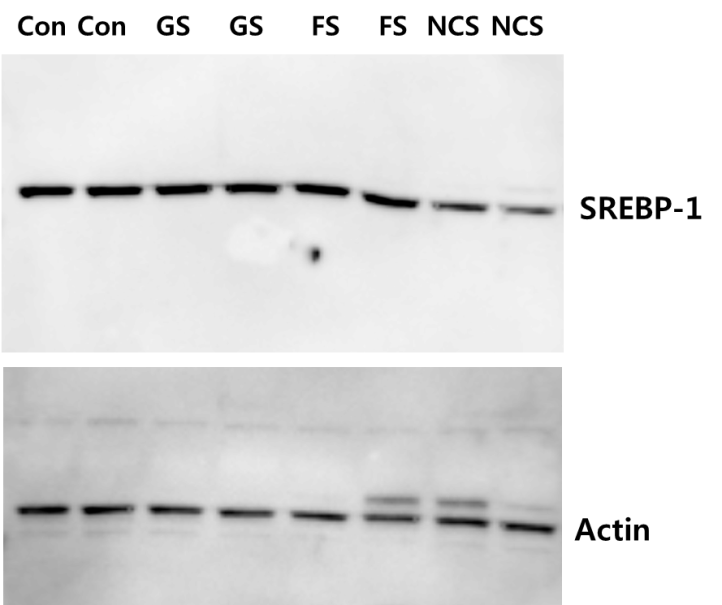


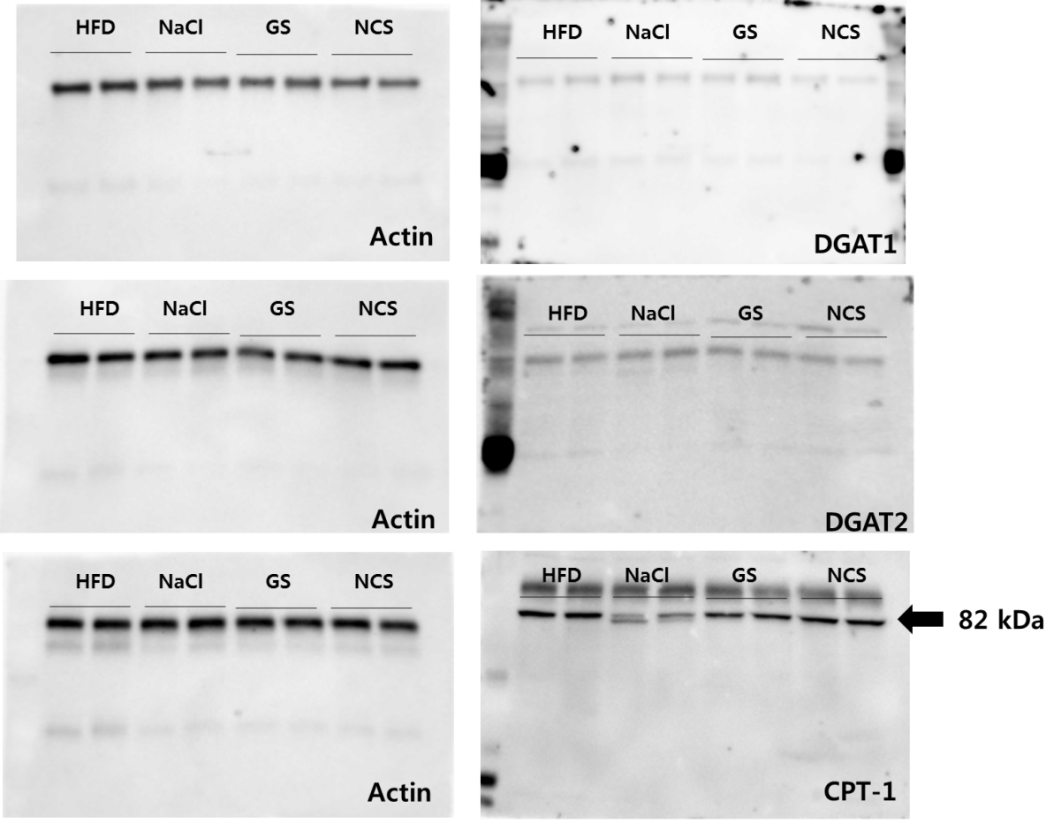


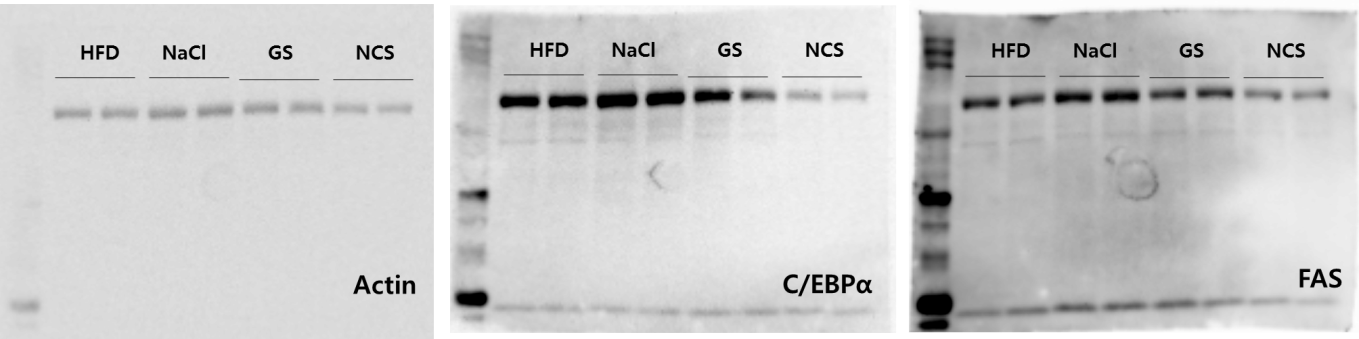


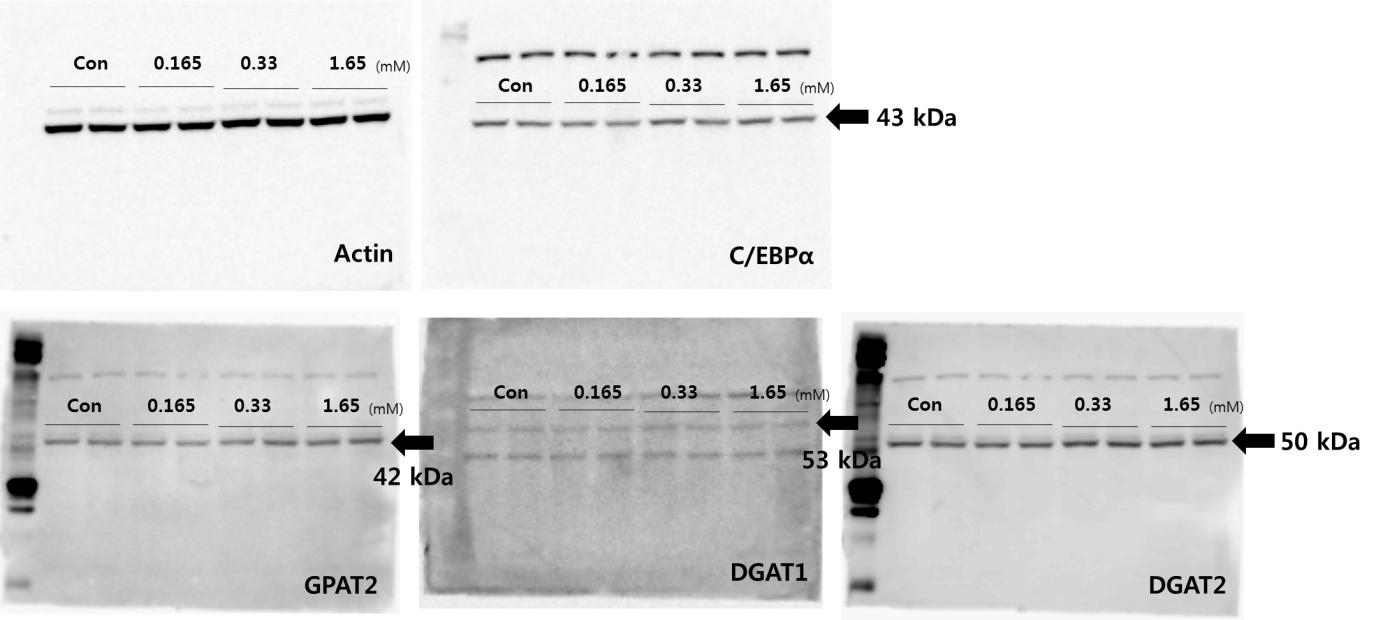


**References**

1. Choi, J. H. Cheonilyeom story in Korea. *Sigma books,* Seoul, p.173-177 (2011).

2. Ha, J. O. & Park, K. Y. Comparison of mineral contents and external structure of various salts. *J. Korean Soc. Food Sci. Nutr.* **27**, 413-418 (1998).

3. Ju, J., Kim, Y. J., Park, E. S. & Park, K. Y. Korean solar salt ameliorates colon carcinogenesis in an AOM/DSS-induced C57BL/6 mouse model. *Prev. Nutr. Food Sci.* **22**, 149-155 (2017).

4. Ju, J., Song, J. L., Park, E. S., Do, M. S. & Park, K. Y. Korean sea salts reduce obesity and alter its related markers in diet-induced obese mice. *Nutr. Res. Pract.* **10**, 629-634 (2016).

5. Pym, R. A. E. & Nicholls, P. J. Selection for food conversion in broilers: Direct and correlated responses to selection for body‐weight gain, food consumption and food conversion ratio. *British Poultry Sci*. **20**, 73-86 (1979).

6. Fischer, A. H., Jacobson, K. A., Rose, J. & Zeller, R. Hematoxylin and eosin staining of tissue and cell sections. *Cold Spring Harb. Protoc.* doi:10.1101/pdb.prot4986 (2008).
